# *Coccidioides* undetected in soils from agricultural land and uncorrelated with time or the greater soil fungal community on undeveloped land

**DOI:** 10.1371/journal.ppat.1011391

**Published:** 2023-05-25

**Authors:** Robert Wagner, Liliam Montoya, Jennifer R. Head, Simon Campo, Justin Remais, John W. Taylor

**Affiliations:** 1 Department of Plant & Microbial Biology, University of California Berkeley, Berkeley, California, United States of America; 2 Division of Epidemiology, University of California Berkeley, Berkeley, California, United States of America; 3 Division of Environmental Health Sciences, University of California Berkeley, Berkeley, California, United States of America; Max-Planck-Institut fur Evolutionsbiologie, GERMANY

## Abstract

Coccidioidomycosis is a typically respiratory fungal disease that, in the United States, occurs primarily in Arizona and California. In California, most coccidioidomycosis cases occur in the San Joaquin Valley, a primarily agricultural region where the disease poses a risk for outdoor workers. We collected 710 soil samples and 265 settled dust samples from nine sites in the San Joaquin Valley and examined how *Coccidioides* detection varied by month, site, and the presence and abundance of other fungal species. We detected *Coccidioides* in 89 of 238 (37.4%) rodent burrow soil samples at five undeveloped sites and were unable to detect *Coccidioides* in any of 472 surface and subsurface soil samples at four agricultural sites. In what is the largest sampling effort undertaken on agricultural land, our results provide no evidence that agricultural soils in the San Joaquin Valley harbor *Coccidioides*. We found no clear association between *Coccidioides* and the greater soil fungal community, but we identified 19 fungal indicator species that were significantly associated with *Coccidioides* detection in burrows. We also did not find a seasonal pattern in *Coccidioides* detection in the rodent burrow soils we sampled. These findings suggest both the presence of a spore bank and that coccidioidomycosis incidence may be more strongly associated with *Coccidioides* dispersal than *Coccidioides* growth. Finally, we were able to detect *Coccidioides* in only five of our 265 near-surface settled dust samples, one from agricultural land, where *Coccidioides* was undetected in soils, and four from undeveloped land, where *Coccidioides* was common in the rodent burrow soils we sampled. Our ability to detect *Coccidioides* in few settled dust samples indicates that improved methods are likely needed moving forward, though raises questions regarding aerial dispersal in *Coccidioides*, whose key transmission event likely occurs over short distances in rodent burrows from soil to naïve rodent lungs.

## Author summary

Valley fever (coccidioidomycosis) is a fungal disease of humans that, in California, most commonly occurs in the San Joaquin Valley. Valley fever is caused by infection with *Coccidioides* fungi, of which nearly all cases occur though inhalation of airborne spores. Many cases of Valley fever in the San Joaquin Valley occur amongst agricultural workers, though only one other study has attempted to detect *Coccidioides* in agricultural soils. Furthermore, Valley fever cases follow a seasonal pattern, though no other work has determined whether the abundance of *Coccidioides* fungi in the environment follows a similar pattern. Here, we undertake what is the largest survey of *Coccidioides* amongst agricultural soils. Additionally, we provide the most comprehensive monthly survey of *Coccidioides*, spanning an entire year, amongst soils in the natural environment, namely, within rodent burrows, an environment where *Coccidioides* is commonly found. We were unable to detect *Coccidioides* in any of the agricultural soils we sampled, nor did we find a seasonal pattern in *Coccidioides* detection in the rodent burrow soils we sampled on undeveloped land. Finally, we found few associations between *Coccidioides* and other soil fungi, and no significant association with the structure of the greater soil fungal community. Taken together, our findings challenge assumptions regarding the prevalence of *Coccidioides* in agricultural soils and the relative importance of seasonal growth versus dispersal of *Coccidioides* in the environment.

## Introduction

Over the past two decades, the annual reported number of cases of the fungal disease, coccidioidomycosis (also known as “Valley fever” and, historically, as “desert rheumatism”), has increased approximately 8-fold in the United States [1]. Estimates from 1990 through 2016 show that there was an average of 172–200 coccidioidomycosis associated deaths per year in the United States, with thirty three percent of deaths occurring in Arizona and forty seven percent in California [1,2]. Annually, coccidioidomycosis is estimated to cost approximately $3.9 billion in the United States, and this cost is predicted to increase to as much as $18.5 billion by 2090 [3]. Coccidioidomycosis is most commonly caused by inhalation of arthroconidia (spores) from the fungus *Coccidioides* [4], and individuals engaged in outdoor occupations, particularly those that disturb soils, experience an elevated risk of exposure [5–7]. In California, agriculture employs approximately 800,000 workers, the largest group of outdoor workers in the state [8], and one that is commonly associated with soil disturbance [9,10]. Nearly half of the agricultural workers in California are employed in the San Joaquin Valley (SJV) [8], home to 4.2 million people and the most economically important agricultural region in the United States [11,12]. The SJV is also where the majority of statewide coccidioidomycosis cases are reported [13]. While most cases of coccidioidomycosis fully resolve, those that are chronic or disseminated require life-long treatment with antifungal drugs [14,15]. As there is no cure or vaccine for coccidioidomycosis [16], prevention is paramount, and a better understanding of the distribution of *Coccidioides* in the environment is needed to inform strategies to prevent infection. Our previous comparison of fungal communities from agricultural and undisturbed soils used metabarcoding, which rarely detected *Coccidioides* [17]. Here we combine a sensitive qPCR method of detecting *Coccidioides* with our data on fungal communities to investigate the presence of *Coccidioides* in soil and air in the SJV on both agricultural and undeveloped land (which we define as generally uninhabited land showing no evidence of recent cultivation or irrigation). Given the seasonal fluctuations in the incidence of coccidioidomycosis [16,18,19], our investigation of *Coccidioides* is longitudinal, examining *Coccidioides* detection spanning three summer seasons on agricultural land and 12 months within a full year on undeveloped land. Lastly, we search for relationships between *Coccidioides* and the structure of the greater fungal community.

Coccidioidomycosis is caused by species of *Coccidioides* fungi in the Onygenales. It was first described as a protozoan disease in Argentina in 1892 [20], and later shown to be a fungal disease in California in 1905 [4]. Two species of *Coccidioides* have been identified, *C*. *immitis* and *C*. *posadasii* [21]. *C*. *immitis* is reported from Washington State through the southern SJV in California and into northern Mexico, while *C*. *posadasii* is found from Arizona east to Texas, as well as parts of Mexico, Central America, and South America [22–27]. *Coccidioides* is thought to live as both a saprotroph as well as an animal parasite [16,28,29]. It is commonly found in association with mammals, for example, in soils near carcasses of experimentally infected dogs and mice [30], directly from rodents [31,32], and in and around rodent burrows [33,34]. Importantly, *Coccidioides* is detected more commonly from soils within rodent burrows than from surrounding surface soils, providing evidence for a relationship between *Coccidioides*, rodents and the microhabitat within rodents burrows [34]. *Coccidioides* genome, when compared to the genomes of fungi known to consume plant material, shows a reduction in genes that code for plant cell wall degrading enzymes and an increase in protease encoding genes [35]. *Coccidioides* is known to grow on keratin [34], and another, closely related member of the Onygenales, *Uncinocarpus ressi*, has been shown to favor peptides over carbohydrates as a food source (Desjardins et al. 2011).

### *Coccidioides* in agricultural soils

Agricultural workers constitute the largest group of outdoor workers in California and there is evidence that they are at increased risk for coccidioidomycosis, even as many cases likely go unreported [36,37]. Between 2000 and 2007, the highest percentage of worker compensation claims for coccidioidomycosis in California, five percent (23 of 461), was in the broad occupational category, “Farming, fishing and forestry” [5]. More recently, a case control study of 110 Hispanic farmworkers who had tested positive for *Coccidioides* exposure between June 2016 and August 2018 in Kern County, SJV [38] found that those who reported working with root crops were significantly more likely to have had a positive *Coccidioides* exposure tests than those who reported performing leaf removal tasks, implying that working more intimately with agricultural soil may increase ones risk of *Coccidioides* infection. Importantly, McCurdy et al. (2020) admit that their sample size limited statistical power. However, no coccidioidomycosis outbreaks (≥2 simultaneous infections) have been reported on agricultural land, whereas many outbreaks have been reported on undeveloped land known to harbor *Coccidioides*, nearly always following soil excavation [7,39,40]. Furthermore, *Coccidioides* has never been detected in agricultural soils, though it has been detected numerous times on undeveloped and non-agricultural land throughout the SJV [41–47]. Only one of these studies, however, compared agricultural land with nearby land that is not in agricultural production [47]. This study was unable to detect *Coccidioides* in soil samples from 13 agricultural fields in the SJV, near Lemoore, CA, using a nested PCR approach that detected *Coccidioides* in five soil samples collected on nearby undeveloped lands and adjacent roads [47]. Given these findings, we propose the following hypothesis: *Coccidioides* will be undetectable in agricultural soils when using the same methods that routinely detect *Coccidioides* in rodent burrow soils on undeveloped land, an environment where *Coccidioides* is commonly found. If we can detect *Coccidioides* in soils on agricultural land, and our hypothesis can be falsified, our study would provide experimental support for the notion that agricultural soils in the SJV can harbor *Coccidioides*. However, if our hypothesis cannot be falsified, then the perception that agricultural soils in the SJV are likely to harbor *Coccidioides* will continue to lack an experimental basis.

### *Coccidioides* seasonality

Coccidioidomycosis incidence is associated with seasonality in both Arizona and California, with incidence peaking biannually (early summer and late fall) in Arizona and annually (fall) in California [16,18,19]. In Arizona, it is hypothesized that *Coccidioides* grows in soil during wet periods, followed by spore dispersal during dry periods, leading to increased incidence of coccidioidomycosis [19,48,49]. Similar but weaker evidence has been found for this relationship in California [50,51]. However, in California, drought may also affect coccidioidomycosis seasonal dynamics. Coccidioidomycosis incidence, especially during fall months, is suppressed during drought periods, and amplified in the two years that follow, with over 80% of the drought-attributable excess cases estimated to occur during September through November [52]. Coccidioidomycosis, however, requires inhalation of airborne spores by a susceptible host, and the concentration of *Coccidioides* spores in the outdoor air environment depends both on the aerosolization of soil and the concentration of *Coccidioides* in this soil. In neither Arizona nor California has detection of *Coccidioides* in soils been used to inform hypotheses regarding the mechanism by which coccidioidomycosis seasonality occurs, with the untested assumption being that the abundance of *Coccidioides* in soil reflects seasonal trends in coccidioidomycosis incidence. As a result, it is unclear to what degree the documented seasonal fluctuations in coccidioidomycosis incidence are driven by seasonal changes in *Coccidioides* concentrations in soils or the aerosolization of such soils. To address this knowledge gap, and provide the first study of *Coccidioides* seasonality in soils, we use the sensitive CocciENV qPCR assay to analyze our 238 rodent burrow soil samples collected monthly for one year. We hypothesize that, when controlling for spatial and environmental variables, the presence of *Coccidioides* in soil will vary temporally and this variation can be described in terms of coccidioidomycosis seasonality.

### Interactions with other fungi

Finally, we examine *Coccidioides* presence in the context of the greater soil fungal community using metabarcoding of fungal ribosomal DNA, leveraging recent work we undertook investigating relationships between the soil and airborne mycobiomes in the SJV [17]. Early studies comparing *Coccidioides* with other soil microbes are scant and have focused on individual taxa that inhibit the growth of *Coccidioides*. These taxa include the fungus *Penicillium janthinellum* and the bacterium *Bacillus subtilis*, which, when isolated from soil, inhibit *Coccidioides* growth in culture [52]. More recently, bacteria cultivated from soil that are related to *Bacillus subtilis* and *Streptomyces* spp. were similarly shown to inhibit *Coccidioides* growth in culture [53]. However, other studies have isolated Gymnoascaceae, *Chrysosporium* and *Malbranchia* (genera that share the Onygenales order with *Coccidioides*) from soil along with *Coccidioides*, hinting at possible co-occurrence patterns [43,54]. We are aware of one other study that used metabarcoding to relate *Coccidioides* with the *soil* fungal community across 15 soil (non-rodent burrow) samples from Venezuela [55], finding correlations between *Coccidoides* presence and six other fungal taxonomic groups (Chytridiomycetes, Chaetothyriales, Pleosporales, Dothioraceae, Trichoderma and Ajellomyces). Finally, there is recent research comparing *Coccidioides* and the fungal community within rodent lungs [56] using a metabarcoding approach, finding no significant association between the two. In light of the paucity of ecological analyses comparing *Coccidioides* with other members of the soil fungal community, our analyses in this regard remain exploratory in nature.

## Results

**Fig 1:**
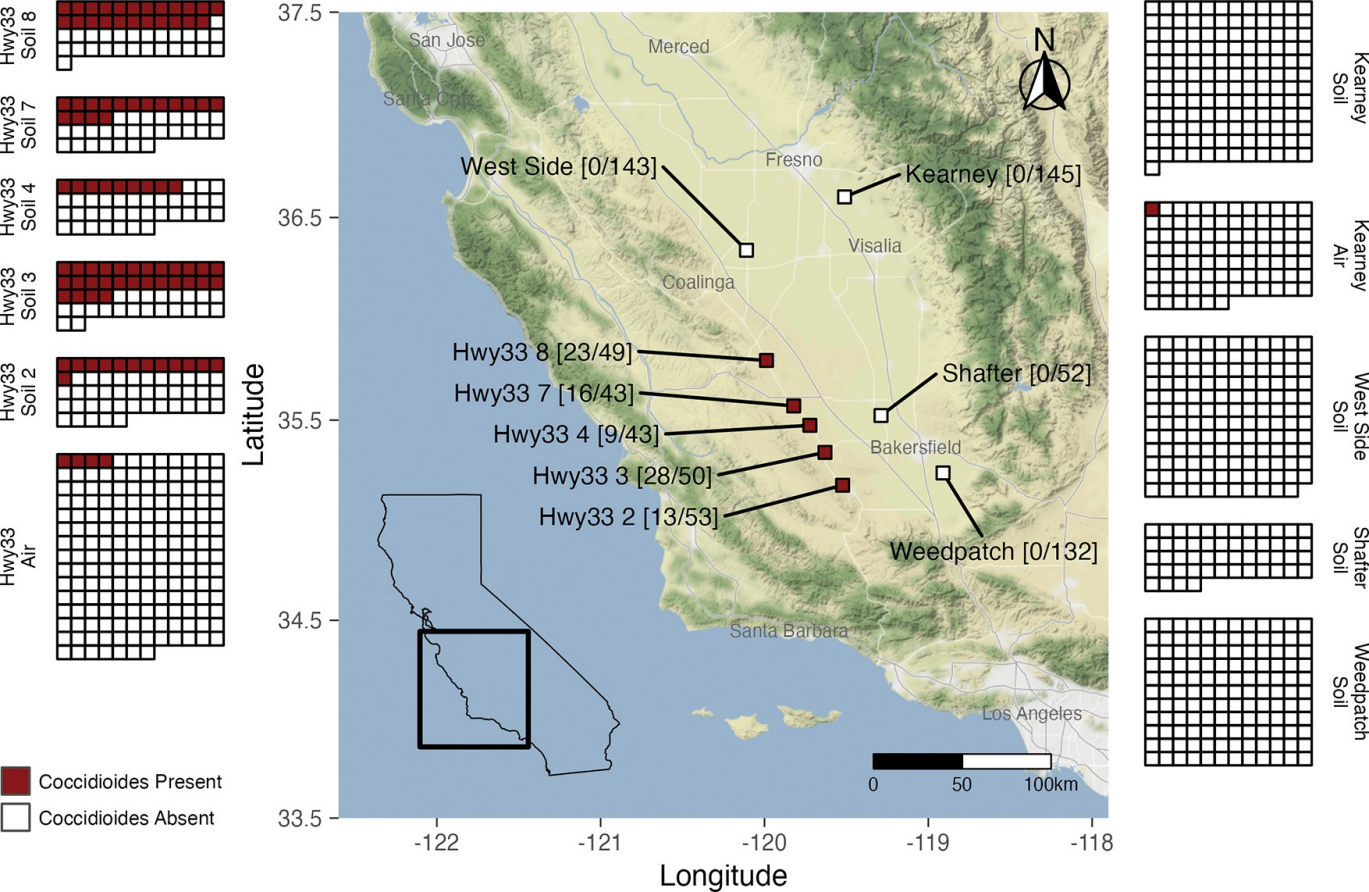
Sampling locations in the San Joaquin Valley in California with *Coccidioides* detection using the CocciEnv qPCR assay depicted for each individual soil or air (settled dust) sample. Numbers in square brackets on map show soil samples positive for *Coccidioides* detection over the total number of soil samples collected at each site. Grids enumerate positive and negative samples at each site. Hwy33 = undeveloped sites. West Side, Kearney, Shafter and Weedpatch = agricultural sites. All soils from Hwy33 sites are from rodent burrows. All soils from agricultural sites are from agricultural fields. Positive Hwy33 air samples are from site 8 (3 samples) and site 3 (1 sample). Total samples across all sites = 975. Map tiles by Stamen Design (www.stamen.com) under Creative Commons By Attribution (CC BY 3.0) and map data by OpenStreetMap (www.openstreetmap.org) under the Open Data Commons Open Database License (ODbL). California shapefile is public domain data by Natural Earth (www.naturalearthdata.com).

We collected a total of 975 samples across nine sites spanning the San Joaquin Valley, including 472 soil samples from agricultural land (Kearney, West Side, Shafter and Weedpatch), 90 settled dust samples from agricultural land (Kearney), and 238 soil samples from rodent burrows and 175 settled dust samples from undeveloped land at five sites along Hwy33. All samples were tested for *Coccidioides* using the CocciEnv qPCR assay (Fig 1) and fungal communities had been characterized for all Hwy33 and Kearny samples using ITS2 barcoding [17,57,58].

### *Coccidioides* detection in soils and settled dust

*Coccidioides* was detected (qPCR positive in ≥ 6 of 8 replicate wells) in soils from rodent burrows from all five undeveloped sites along Hwy33 and was not detected in any agricultural soils from the four agricultural sites investigated (qPCR positive in ≤ 2 of 8 wells, on just one of two replicate plates) (Table 1). CocciENV qPCR analysis of samples from sites along Hwy33 found 89 (37.4%) positive rodent burrow soil samples and 149 (62.6%) negative rodent burrow soil samples. Of the 149 negative samples, 102 (68.4%) showed no detection in any well, and of the remaining 47 samples, 27 showed positive wells on both replicate plates and could potentially represent false negatives. Among the 472 agricultural soil samples, there were no samples that met the criteria for positivity. Of these 472 negative samples, 432 (91.5%) showed no detection in any well. The 40 remaining samples were positive in ≤ 2 replicate wells and only on one of the two, replicate qPCR plates. Among the 265 settled dust samples we investigated, *Coccidioides* was detected (qPCR positive in ≥ 3 of 4 replicate wells) in four samples from undeveloped sites along Hwy33 and one sample from the Kearney agricultural site. Three of the settled dust samples from Hwy33 sites were positive in 4/4 wells, a fourth was positive in 3/4 wells and there were four additional Hwy33 settled dust samples that did not meet the criteria for positivity that showed detection ≤ 2 wells. The single positive settled dust sample collected at the Kearney agricultural site showed detection in 4/4 wells and no other settled dust sample collected at Kearney showed detection in any well. *Coccidioides* detection using ITS2 metabarcoding from sites along Hwy33 found 4 positive rodent burrow soil samples, as previously described [17], all of which had tested positive using the CocciEnv qPCR assay. *Coccidioides* was not detected using ITS2 metabarcoding from any of the agricultural soil samples from Kearney, nor from any settled dust sample, whether from Hwy33 sites or Kearney, via ITS2 metabarcoding.

**Table 1:** *Coccidioides* detection level reported as the number of reactions testing positive at CT < 40 with logarithmic amplification using the CocciEnv qPCR assay. Soil samples considered positive for *Coccidioides* are enumerated in the first two columns and include samples with 8/8 positive wells and samples with either 6/8 or 7/8 positive wells. Samples considered negative for *Coccidioides* are enumerated in the next four columns and include all samples with < 6 positive wells. Note: settled dust samples used a more liberal criterion for positivity whereby samples with either 4/4 or 4/3 positive wells were considered positive for *Coccidioides* and are enumerated in the first two columns and samples considered negative for *Coccidioides* include all samples with < 3 positive wells and are enumerated in the following three columns. Numbers in parentheses in column headings indicate the number of positive wells for each detection level. * indicates detection level derived from a single composite (transect) soil sample (total subsamples from transect in numerator) from a single qPCR assay. Soil samples from Kearney soil cores in the Negative (2–3) category tested positive in no more than two of eight wells and from only one of the two, replicate qPCR test plates.

	Positive (8)	Positive (6–7)	Negative (4–5)	Negative (2–3)	Negative (1)	Negative (0)	Total
Hwy33 8 Burrow Soil	17	6	2	6	0	18	49
Hwy33 7 Burrow Soil	12	4	2	4	2	19	43
Hwy33 4 Burrow Soil	8	1	1	4	1	28	43
Hwy33 3 Burrow Soil	23	5	9	4	4	5	50
Hwy33 2 Burrow Soil	10	3	1	2	5	32	53
	**70**	**19**	**15**	**20**	**12**	**102**	**238**
	Positive (8)	Positive (6–7)	Negative (4–5)	Negative (2–3)	Negative (1)	Negative (0)	Total
Kearney Soil Core	0	0	0	3	12	130	145
West Side Surface Soil	0	0	0	≥ 1/13 *	0	130	143
Shafter Surface Soil	0	0	0	0	1	51	52
Weedpatch Surface Soil	0	0	0	0	≥ 1/11 *	121	132
	**0**	**0**	**0**	**4–16**	**14–24**	**432**	**472**
	Positive (4)	Positive (3)	Negative (2)	Negative (1)	Negative (0)	Total	
Hwy33 8 Settled Dust	2	1	0	0	31	34	
Hwy33 7 Settled Dust	0	0	0	1	33	34	
Hwy33 4 Settled Dust	0	0	1	1	34	36	
Hwy33 3 Settled Dust	1	0	0	1	34	36	
Hwy33 2 Settled Dust	0	0	0	0	35	35	
Kearney Settled Dust	1	0	0	0	89	90	
	**4**	**1**	**1**	**3**	**256**	**265**	

### *Coccidioides* seasonality and site differences

**Fig 2:**
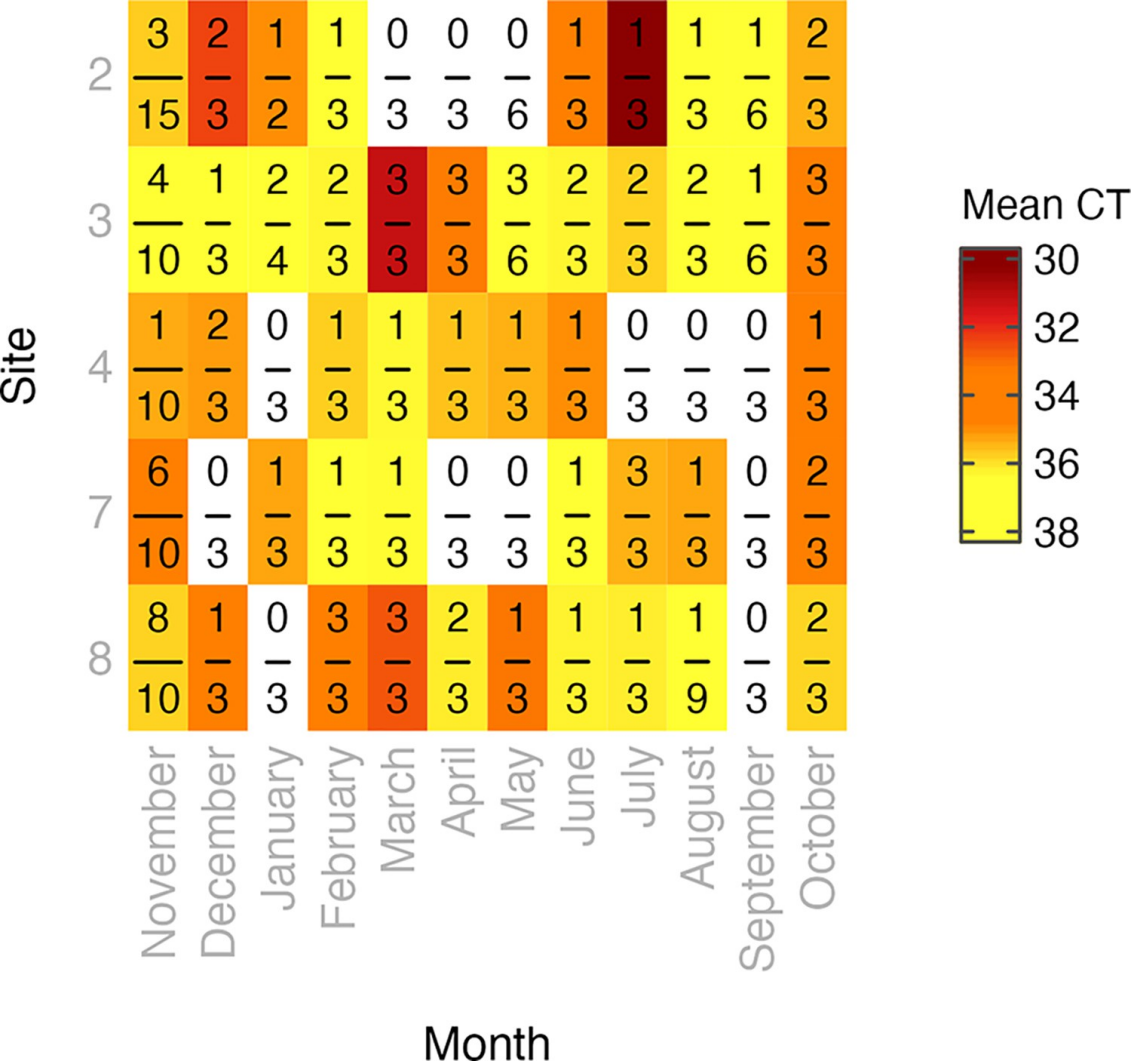
Detection of *Coccidioides* using the CocciEnv qPCR assay as a function of month and site from Hwy33 burrow soil samples. Numerator = burrow soil samples positive for detection of *Coccidioides*. Denominator = total burrow soil samples collected. Gray cells = samples negative for *Coccidioides*. CT = qPCR cycle threshold value. Mean CT value range = 30.3–38.0. n = 238.

**Fig 3:**
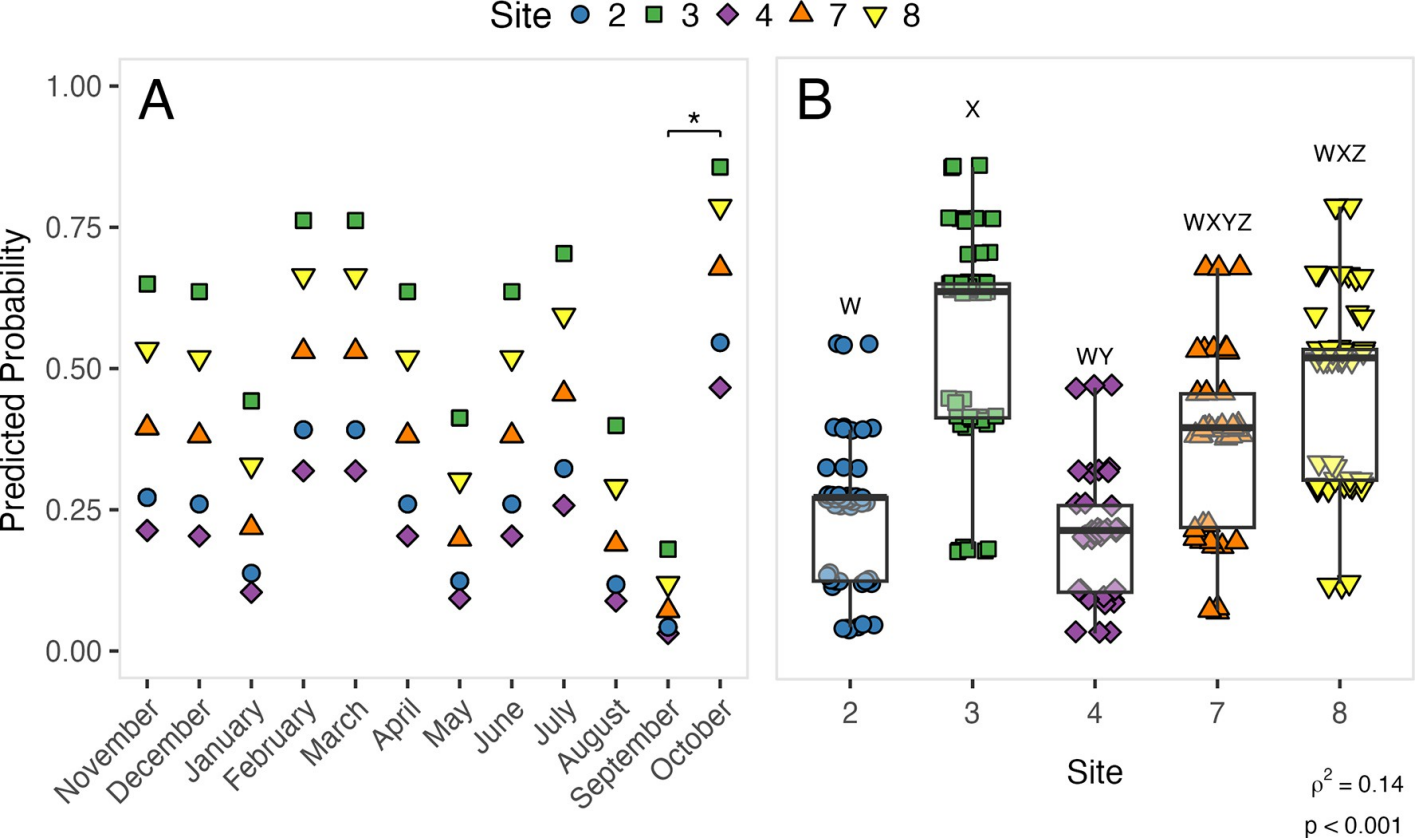
The predicted probability (derived from logistic regression) of detecting *Coccidioides* as a function of month and site. Predicted probabilities are displayed as means as a function of month (A) and as individual samples as a function of site (B) (points jittered for clarity). * = months differ significantly (* = p < 0.05, ** = p < 0.01, *** = p ≤ 0.001, Tukey-adjusted pairwise contrasts). Letters (XYZ) denote groups of sites that do not significantly differ (p > 0.05, Tukey-adjusted pairwise contrasts). Total month contrasts = 66. Total site contrasts = 10. “rho squared” (ρ^2^) = McFadden’s pseudo r^2^. p = significance of full logistic regression model derived from deviance and null deviance. n = 238.

A positive CocciENV qPCR *Coccidioides* signal was detected in soils at all 5 sites and in all 12 months investigated along Hwy33 (Fig 2). There was no clear pattern in the probability of detecting *Coccidioides* across sampling months (Fig 3A), with the only significant difference observed (mean ± standard error of the mean (SEM)) between September and October (p = 0.03), at, respectively, 0.10±0.01 and 0.67±0.04. Site differences were pronounced, however, with the probability of detecting *Coccidioides* significantly higher at sites 3 (0.56±0.03) and 8 (0.47±0.02) than at sites 2 (0.25±0.02) and 4 (0.21±0.02) (Fig 3B). The probability of detecting *Coccidioides* at Site 7 (0.37±0.02) did not significantly differ from that at sites 2, 3, 4, and 8. Notably, the four *Coccidioides* detections in settled dust from Hwy33 sites were from sites 3 (1 sample) and 8 (3 samples). Adjusting for time-varying environmental covariates in the model, which had little spatial variation, did not significantly affect coefficients on month and site (S2 Fig and S3 and S4 Tables). In both the simple and fully adjusted models, the probability of detecting *Coccidioides* remained largely unassociated with the timing of sample collection and continued to exhibit clear and significant differences between sites (Tables 2 and 3). Model specification using harmonic or spline transformations of month did not significantly change our results, and the fit of both approaches to our data was significantly worse (ΔAIC = +11.1 and +15.7, respectively) than treating sampling month as a categorical variable (S3 Fig and S5 Table).

**Table 2:** Logistic regression coefficient table showing (using the “glm” function) positive *Coccidioides* detection using the CocciEnv qPCR assay, as a function of sampling site and sampling month. n = 238.

	Estimate	Standard Error	z-value	p-value	
Intercept	-1.835	0.702	-2.613	0.009	**
February	1.394	0.826	1.689	0.091	.
March	1.394	0.826	1.689	0.091	.
April	0.789	0.83	0.952	0.341	
May	-0.123	0.815	-0.15	0.88	
June	0.789	0.83	0.952	0.341	
July	1.095	0.825	1.327	0.184	
August	-0.179	0.814	-0.22	0.826	
September	-1.286	0.977	-1.316	0.188	
October	2.018	0.847	2.382	0.017	*
November	0.849	0.682	1.245	0.213	
December	0.789	0.83	0.952	0.341	
Site 3	1.604	0.463	3.461	< 0.001	***
Site 4	-0.319	0.514	-0.621	0.535	
Site 7	0.561	0.472	1.187	0.235	
Site 8	1.121	0.46	2.435	0.015	*

. = p < 0.1

* = p < 0.05

** = p < 0.01

*** = p ≤ 0.001

**Table 3:** Pairwise differences between sites and, independently, between months by comparing estimated marginal means between logistic regression factor levels. Logistic regression calculated as positive *Coccidioides* detection using the CocciEnv qPCR assay, as a function of sampling site and sampling month. Only significant contrasts are shown. p-values are Tukey adjusted to total contrasts performed. Total month contrasts = 66. Total site contrasts = 10.

Contrast	Estimate	Standard Error	z-value	p-value	
Site 2 –Site 3	-1.604	0.463	-3.461	0.005	**
Site 3 –Site 4	1.923	0.508	3.788	0.001	**
Site 4 –Site 8	-1.44	0.502	-2.867	0.034	*
Sept–Oct	-3.304	0.965	-3.422	0.031	*

. = p < 0.1

* = p < 0.05

** = p < 0.01

*** = p ≤ 0.001

### Soil fungal community

**Fig 4:**
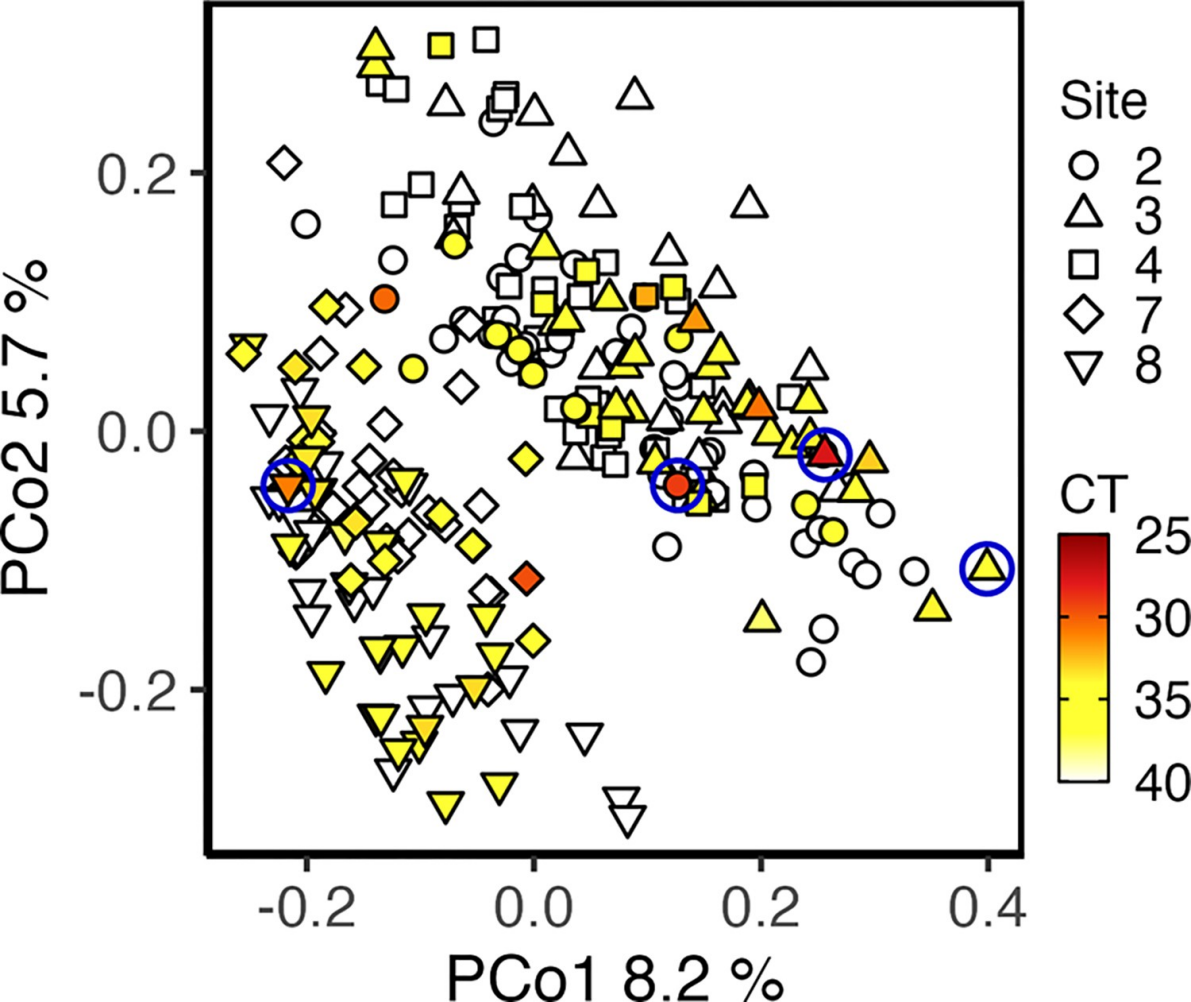
PCoA plot showing the Bray-Curtis dissimilarity within the fungal community between individual rodent burrow soil samples from Hwy33 sites. Colored points = samples positive for *Coccidioides* using the CocciENV qPCR assay. White points = samples negative for *Coccidioides* using the CocciENV qPCR assay. Blue circles = samples positive for *Coccidioides* in the ITS2 rDNA dataset. CT = qPCR cycle threshold value. n = 238.

The soil fungal community composition was associated with both site and month but not meaningfully associated with *Coccidioides* detection. The relationship between *Coccidioides* detection and soil fungal community β-diversity from Hwy33 rodent burrows, as represented through Bray-Curtis dissimilarity, was trivial (r^2^ = 0.01) although statistically significant (p = 0.004). Instead, the soil fungal community composition was more strongly associated with sampling site (r^2^ = 0.17, p = 0.001), and to a lesser extent, sampling month (r^2^ = 0.08, p = 0.001), as previously reported [17] (Fig 4 and Table 4). Adjusting for environmental covariates did not meaningfully change the relationship between *Coccidioides* detection and the composition of the soil fungal community (S6 Table).

**Table 4:** PERMANOVA results showing the ITS2 rDNA derived fungal community as a function of positive *Coccidioides* detection using the CocciEnv qPCR assay (Presence), sampling site and sampling month, from Hwy33 rodent burrow soil samples. Permutations = 1000. n = 238.

	Degrees of Freedom	Sum of Squares	r^2^	Pseudo-F	p-value	
*Coccidioides* Detection	1	0.29	0.01	1.91	0.004	**
Site	4	8.03	0.17	13.29	0.001	***
Month	11	3.72	0.08	2.24	0.001	***
*Coccidioides* Detection: Site	4	0.80	0.02	1.33	0.006	**
*Coccidioides* Detection: Month	11	1.69	0.04	1.02	0.377	
Site: Month	44	8.50	0.18	1.28	0.001	***
*Coccidioides* Detection: Site: Month	25	3.40	0.07	0.90	0.990	
Residual	137	20.69	0.44			
**Total**	**237**	**47.13**	**1**			

. = p < 0.1

* = p < 0.05

** = p < 0.01

*** = p ≤ 0.001

To account for the possibility that the most abundant fungal species obscure relationships between *Coccidioides* and less abundant fungi, we repeated our PERMANOVA analyses after removing the ten most abundant species (S4A Fig and S7 Table) and after removing the 50 most abundant species (S4B Fig and S8 Table). The most common fungal species identified in Hwy33 rodent burrow soil communities generally are, or belong to groups known to be, parasites of crops and wild plants (S5 Fig). These taxa include *Didymella* spp. [59,60], *Coniochaeta* spp. [61] *Mycosphaerella* spp [62] and *Alternaria* spp. [63–65], which can be associated with seeds and grains cached in burrows of rodents living in arid environments [66–68]. Removing abundant species made little difference in the relationship between *Coccidioides* and the fungal community when compared to the full dataset. Inclusion of environmental covariates in PERMANOVA analyses of the reduced taxonomic datasets did not change our results (S9 and S10 Tables).

Our indicator species analysis identified eight fungal OTUs in our ITS2 sequencing data set that significantly co-occurred with *Coccidioides* detection using the CocciENV qPCR assay in the same rodent burrow soil samples. We also identified two fungal OTUs that were significantly associated with rodent burrow soil samples lacking *Coccidioides* detection. Our further analysis of the sequences associated with these OTUs revealed 16 likely fungal indicator species co-occurring with *Coccidioides* and three fungal indicator species not co-occurring with *Coccidioides* (S11 Table). *Aspergillus penicillioides*, a xerophilic and halophilic fungus with a global distribution [69,70], was strongly (IndVal = 0.61) and significantly (p = 0.001) associated with *Coccidioides*-positive samples. This fungus is also associated with stored grain and may be found in rodent food caches [71]. Other species that were associated with *Coccidioides*-positive samples and showed a high degree of significance (p < 0.01) included: *Acremonium acutatum*, a *Vitis* (grape) parasite [72]; *Fusarium spp*., a widespread genus commonly associated with soils and plants, but one that also includes some opportunistic pathogens of animals [73]; *Tricellula inaequalis*, a member of a genus with the spore morphology of aquatic sporulating, leaf endophytes [74–76]; *Tulostoma pseudopulchellum*, a basidiomycete stalked “puffball” that is frequently observed in arid regions and whose spores are wind-dispersed from an elevated chamber [77]; and *Simplicillium album*, whose genus is associated with parasitism of plants and animals and saprotrophy of decaying animal substrates [78]. Reassuringly, *Coccidioides* detected in the ITS2 sequencing data set was also significantly associated with samples positive for *Coccidioides* using the CocciENV qPCR assay (p = 0.02). The three indicator species associated with rodent burrow soils lacking *Coccidioides* were all members of the Spizellomycetales, a fungal order of flagellated, aquatic fungi containing saprobic and parasitic species, including several known to parasitize the spores of other fungi [79–81].

## Discussion

In the present study, we demonstrated a high probability of detecting *Coccidioides* in soils from rodent burrows on undeveloped land and, using the same protocol, an inability to reliably detect *Coccidioides* in surface and subsurface soils from agricultural land in the San Joaquin Valley in California. We showed this difference by testing 238 rodent burrow soil samples from five sites on undeveloped land and 472 surface and subsurface soil samples from four sites on agricultural land. We did not find a clear temporal pattern in *Coccidioides* detection in rodent burrows soils at our five undeveloped sites along Hwy33 in Kern and Kings Counties, though we did find meaningful spatial variation between these sites. We found little evidence for a relationship between *Coccidioides* and the structure of the greater soil fungal community, though significant co-occurrence patterns between *Coccidioides* and up to sixteen fungal species were identified, as well as significant patterns of an absence of co-occurrence between *Coccidioides* and three fungal species. Finally, we detected *Coccidioides* in five of the 265 settled dust samples we collected, four from undeveloped land where we detected *Coccidioides* in rodent burrow soils, and one from agricultural land where we were unable to detect *Coccidioides* in soils.

### *Coccidioides* in agricultural soils

Our most pertinent finding was our inability to convincingly detect *Coccidioides* in any of the 472 soils we collected from four agricultural sites in three counties (Kern, Kings and Fresno) in the SJV, leaving intact our hypothesis that *Coccidioides* would be undetectable in agricultural soils. We cannot claim that *Coccidioides* is not present in any agricultural soil at any time or in amounts below our limit of detection. However, our sampling effort was thorough and included soils from both east and west sides of the SJV (the former being well irrigated and the latter more arid), representing soils from fields cultivated with both row and tree crops as well as uncultivated fields that had been fallowed for both six years and twelve years. Essential to our inability to falsify our hypothesis was evidence that our approach reliably detected *Coccidioides* in rodent burrow soils, an environment where *Coccidioides* has commonly been found [33,34,82–84]. Using the same method that we employed with agricultural soils, more than a third of the 238 rodent burrow soils we collected from five sites on undeveloped land in two counties (Kern and Kings) yielded positive *Coccidioides* results. Our results are consistent with those from another study that could not detect *Coccidioides* in 13 soil samples from agricultural land while successfully detecting *Coccidioides* in 5 soil samples from nearby undeveloped land, all within 30km of our West Side site [47]. Our inability to detect *Coccidioides* on agricultural land may be due to irrigation that places *Coccidioides* at a competitive disadvantage compared to other fungi [16,85,86] and efforts to exclude rodents from agricultural land in California [87,88], which would limit an animal protein nutrient source that *Coccidioides* has evolved to exploit [35].

### *Coccidioides* seasonality

Our detection of *Coccidioides* in rodent burrow soils was unassociated with sampling month and exhibited no clear intra-annual trends. This finding does not support our hypothesis that the presence of *Coccidioides* in soil would vary with time, instead showing few significant differences in the probability of detecting *Coccidioides* in soils between months or over the course of the study year. Additionally, our finding does not reflect observed intra-annual and seasonal trends in coccidioidomycosis incidence [19,50,89,90]. Seasonal variations in coccidioidomycosis incidence were apparent in California during the time period when our study took place, of which approximately 40% of total cases in California were reported in the counties where we detected *Coccidioides* in rodent burrow soils [13,89]. It has been hypothesized that *Coccidioides* grows in soils following periods of heightened precipitation [19]. During the time of our sampling, November 2017—October 2018, there was a clear pattern of precipitation in January and March of 2018, followed by the absence of precipitation during the rest of the year (S1 Fig). If *Coccidioides* growth in soils was related to precipitation patterns, we would have expected to observe an increase in *Coccidioides* detection in the dry months that followed the wet months, which we did not. An implication of the difference we find between the seasonality of reported coccidioidomycosis cases, and the lack of seasonality in our detection of *Coccidioides* in soil, is that coccidioidomycosis cases may be more related to *Coccidioides* dispersal from soils, or increased exposure among human hosts, than *Coccidioides* abundance or growth in soils. Importantly, dispersal and exposure mechanisms need not be limited to environmental phenomena. For example, one such source of exposure may be the proliferation of solar projects in the SJV [91], of which several have been associated with coccidioidomycosis outbreaks [14,39,92], though any construction projects involving soil excavation in the area should also be considered. Comparing the seasonality of such projects and similar anthropogenic soil disturbances with the seasonality of coccidioidomycosis incidence should be investigated in future studies.

### *Coccidioides* and the soil fungal community

We found only weak evidence for a relationship between *Coccidioides* and the composition of the greater soil fungal community, and this relationship remained weak after adjusting for site, sampling month, and environmental covariates. Although our extensive sampling effort returned a statistically significant association between *Coccidioides* and fungal community β-diversity [93], the very low variance explained by this relationship suggests that it is not ecologically meaningful. It is possible that the soil fungal community partitions along environmental gradients that were not captured by remote sensing data, particularly environmental variables that can shift at the intra-site level such as vegetation cover and landscape microtopography, though further experimentation would be required to test this. Additionally, our sequencing method could only detect *Coccidioides* in four rodent burrow soil samples which contrasts with the CocciEnv qPCR assay, which found *Coccidioides* in 89 rodent burrow soil samples and five settled dust samples. This indicates that other fungi that are as rare or rarer than *Coccidioides* were likely excluded from our community analyses. Nonetheless, we did identify several possible indicator species that significantly co-occurred with *Coccidioides*. *Aspergillus penicillioides*, which strongly and significantly co-occurred with *Coccidioides*, is xerophilic and halophilic [70], characteristics that have also been attributed to *Coccidioides* [94]. *Aspergillus* spp. are associated with stored grain and rodent seed caches [71,95], indicating that current or past rodent activity may be greater in burrows where we detected *Coccidioides*. This association is also supported by the significant co-occurrence between *Coccidioides* and *Simplicillium album*, a member of a genus that is associated with saprotrophy of animal hair [78].

Our finding of only a very weak association between the presence of *Coccidioides* in soils and fungal community β-diversity may indicate that we sampled *Coccidioides* spores rather than actively growing mycelia (assuming actively growing members of the soil fungal community are associated with the overall community structure). *Coccidioides* spores can remain viable when stored (in a laboratory) for up to six months under a variety of temperature and moisture conditions [96] and for as many as four years when stored dry at room temperature [45]. Though speculative, the presence of a substantial *Coccidioides* soil spore bank could mask seasonal fluctuations in the production of new spores, helping to explain the lack of seasonality we found in our detection of *Coccidioides*. Such a spore bank could also ensure a continual source of infection for naïve rodent hosts, further masking seasonal trends in *Coccidioides* growth and dispersal. A transcriptomic approach might be used in future studies to distinguish between spores and active mycelium and assess *Coccidioides* activity in soil environments, though RNA targets still need to be identified.

### Airborne *Coccidioides*

We did not detect *Coccidioides* in all but five of our settled dust samples, even though samples were collected only 50cm above the soil surface at our Hwy33 sites, within meters of rodent burrows where *Coccidioides* was found. Importantly, we were only able to detect *Coccidioides* in settled dust samples after concentrating extracted DNA (though DNA concentrations remained 2–3 orders of magnitude lower than for soil samples). This finding indicates that *Coccidioides* could have been present in more of our settled dust samples, and that improved methods of sample collection and DNA extraction are indicated for future studies. Only four previous studies have successfully detected airborne *Coccidioides* [97–100], all of which relied on filtering >10^6^ liters of air. Ajello et al. (1965) assayed for *Coccidioides* by recovery of the fungus following intraperitoneal injection into mice, Daniels et al. (2002) used a qPCR detection approach that targeted the internal transcribed spacer region of fungal rDNA and Chow et al. (2016) used leaf blowers to aerosolize soil, followed by detection of *Coccidioides* using a nested qPCR approach. Gade et al. (2020) sampled 144,000 liters of air at each of 21 sites and assayed for *Coccidioides* using both nested qPCR as well as the CocciENV assay, the same method used here. The disparity between our study and that of Gade et al. (2020) suggests that *Coccidioides* in ambient air may exist at concentrations that are challenging to detect in settled dust, even when sampling is done 50 cm above soils where *Coccidioides* is present, and that an expanded sampling effort is likely needed.

Although we rarely detected *Coccidioides* in settled dust samples, we commonly detected numerous other fungal species, the most common being *Alternaria* spp., *Mycosphaerella* spp. and *Didymella* spp. (S6 Fig) [17]. This disparity may be explained by the relative ability of different fungi to effectively disperse their spores in air. For example, we commonly detected *Aspergillus* spp. in our survey of the outdoor air mycobiome in the SJV [17], and species of this genus have both a highly evolved conidiophore for elevating spores into the active air layer, and a mechanism to produce multiple spores from a single, stem-like cell [69,101,102]. *Coccidioides*, by contrast, makes spores in one of the simplest possible manners, by conversion of alternating hyphal segments into arthroconidia [103], which provides no mechanism to elevate spores for discharge into active air nor any means to produce more than a single spore from each hyphal segment. This physiological difference suggests that spores released from *Coccidioides* hyphae are not specifically adapted to air transport for transmission, and instead may rely on inhalation by near-surface mammals, such as naïve rodent hosts [28]. Nonetheless, our detection of *Coccidioides* in five settled dust samples, previous detections of airborne *Coccidioides*, and the fact that coccidioidomycosis is overwhelmingly caused by inhalation of *Coccidioides* spores, indicates that airborne *Coccidioides* is certainly present in the SJV and other endemic regions, regardless of how incidental this transmission route may be.

Of particular interest was our unambiguous detection of *Coccidioides* from a single settled dust sample on agricultural land at our Kearney site, especially given our inability to detect *Coccidioides* in soil from the same site (or any agricultural site). The other four settled dust samples where we detected *Coccidioides* were collected from Hwy33 sites 3 and 8, sites that had the highest probability of *Coccidioides* detection in soils. These findings raise the possibility that airborne *Coccidioides* on agricultural land may originate from soils on undeveloped land or other, as-yet-unexplored sources. While speculative, this provides a starting point for exploring hypotheses regarding *Coccidioides* dispersal across land-use types, though improved airborne sampling and DNA extraction methods would be required.

### Methodological considerations

Although we were unable to detect *Coccidioides* in agricultural soils, we cannot rule out the presence of *Coccidioides* in agricultural soils at an abundance too low for detection by our methodology. The CocciDx assay has an experimentally determined limit of detection (LOD) of 15 target copies per reaction [104], while the CocciENV assay used here is reported to be three- to four-fold more sensitive [84]. Theoretically, a single *Coccidioides* spore or cell should be detectable, as the target sequence of the CocciENV assay is a repeat region occurring >80 times in the *Coccidioides* genome [104]. However, a comparison of different soil DNA extraction kits demonstrated low efficiency of DNA extraction from spores in soil [105]. These authors only recovered between 0.1–1% of the possible total DNA from soils spiked with *Bacillus cereus* spores, using essentially the same soil DNA extraction kit used here. Assuming DNA is extracted from *Coccidioides* spores in soils at the same efficiency, and all other conditions are ideal, we would need at least five spores in each soil sample to signal a positive detection. However, if DNA extraction is inefficient, and the sensitivity of the CocciENV assay is the same as the CocciDx assay, nearly 200 spores would be needed for positive detection. For reference, *Coccidioides* infection can occur through inhalation of as few as ten spores [106,107]. Taken together, these considerations indicate that an exploration of different DNA extraction techniques for samples containing *Coccidioides* spores may be needed in future studies, including possible pre-treatments of samples, such as the use of proteinases, heat/freeze cycles and additional bead-beating protocols.

Although we cannot rule out *Coccidioides* in agricultural soil, if it is present, it is much less common than in soils from undeveloped land. It is possible that an expanded sampling effort might detect *Coccidioides* in agricultural soils, given that the distribution of *Coccidioides* in non-agricultural soils is sporadic [45,108]. An understated result of our study was our inability to find rodent burrows on agricultural land at West Side, Shafter, Weedpatch and Kearney, which is likely the result of intensive efforts to prevent rodent pest damage to agricultural crops [87,109]. However, given crop damage estimates [110,111], rodents likely inhabit nearby refugia, either on agricultural land employing less intensive pest control or adjacent wildlands. Additional sampling of agricultural soils should focus on breadth (additional sites), depth (additional samples at each site) and attempt to seek out rodent burrow soils that may be present on agricultural land, such as on long-term out of production fields. An important caveat here being that out of production (fallowed) fields are generally “disced” (or “disked”), a process whereby the soil surface is kept devoid of vegetation in preparation of being brought back into production and to prevent weeds from propagating and negatively affecting nearby fields. Indeed, the fallowed fields we sampled at West Side and Shafter presented as extremely arid, desert-like expanses that are unlikely to serve as refugia for local rodent species. Finally, the effectiveness of our soil homogenization methods likely varied based on soil moisture content. Soils from Hwy33 rodent burrows, or from either fallowed agricultural fields or the periphery of agricultural fields where irrigation was less intensive, were dry and mixed easily by inverting collection tubes. Soils from actively cultivated agricultural fields were homogenized by inverting collection tubes and Kearny samples were homogenized by physically mixing samples. Nonetheless, the 472 agricultural soil samples we collected far exceeds both the thirteen agricultural samples previously examined, as well as the number of samples needed for detection if *Coccidioides* is as prevalent in agricultural soils as on undeveloped land. Based on previous studies showing *Coccidioides* in 18.7 percent of rodent burrow soils and 9.9% of non-rodent burrow soils (S1 Table), if *Coccidioides* was present in agricultural soil at the same rate as in non-rodent burrow soil, we would have expected to find 48 of our 472 soil samples positive for *Coccidioides*, rather than none.

*Coccidioides* detection in air is subject to same concerns as in soil regarding limits of detection and sporadicity. For example, in the one study that detected *Coccidioides* in air using an approach similar to ours [100], air was sampled over 45 days at 21 sites in and around Phoenix, AZ, and at 18 of those 21 sites *Coccidioides* was detected on fewer than five of 45 days. Our monthly sampling of passively settled dust for over one year above soil containing *Coccidioides* yielded only five positive samples, indicating that passive settled dust sampling is less effective at collecting *Coccidioides* than active, high-volume air sampling. However, the high-volume air samplers used by Gade et al. (2020) are expensive, costing at least US$7,000 per instrument plus roughly twice that figure for the mobile solar panels needed for remote operation (PSU-3-H, HI-Q Environmental Products, San Diego, CA, USA), limiting replication at the spatial extent examined herein. One way forward may be to leverage pre-existing infrastructure, e.g., particulate filters on air quality monitoring stations, such as those operated by the California Air Resources Board (CARB) (https://ww2.arb.ca.gov/). Given our ability to detect *Coccidioides* in settled dust following DNA concentration, another simpler option may be to improve our DNA extraction efficiency by pre-treating settled dust samples as mentioned above for soil samples, using higher surface-are samplers and concentrating extracted DNA moving forward.

Our detection of *Coccidioides* relied on the highly sensitive and specific CocciENV qPCR assay [84]. Using this method, we were able to detect *Coccidioides* in twenty-fold more soil samples than with our previous sequencing of the ITS2 region of fungal ribosomal DNA [17]. However, as noted in the methods, PCR amplification tends to lose specificity when target template DNA is absent [112], and particularly so when many PCR primers are used, as is the case with the CocciENV qPCR assay. Therefore, our scoring of soil samples positive for *Coccidioides* was conservative (CT < 40 in at least 6 of 8 wells), ensuring that *Coccidioides* was detected on both replicate plates. This approach to scoring left 15 samples, all from Hwy33 rodent burrows, that returned greater than 3 but fewer than six wells with positive signals, all 15 of which were found on both duplicate plates. We repeated our analyses with the inclusion of these 15 possible false negatives but found no significant difference in results (unreported). Of the 472 agricultural soils sampled, 40 showed amplification in at least one well. For these 40 samples, amplification was detected in ≤ 2 wells and never on both PCR plates, suggesting that these wells may be false positives. Therefore, we consider these 40 soils to be true negatives and examples of the loss of PCR specificity when template is absent.

In conclusion, we demonstrated that *Coccidioides* could not be detected in soils at four agricultural sites in the San Joaquin Valley in California using the same detection method that returned high levels of detection in soils from rodent burrows on undeveloped land. We also show that *Coccidioides* presence in rodent burrow soils is unassociated with sampling month, precipitation, and the structure of the greater surrounding soil fungal community. These findings raise the possibility that *Coccidioides* is inactive or otherwise not growing in the rodent burrow soils we sampled, possibly existing as a spore bank, in turn suggesting that coccidioidomycosis seasonality may be more related to *Coccidioides* dispersal than *Coccidioides* growth in soils. We collected several samples from fallowed fields, though a deeper exploration of fallowed fields, especially non-disced land that has been reinhabited by rodents, would present a valuable research avenue for further studies. While *Coccidioides* was unrelated to the structure of the entire fungal community, we identified 16 individual fungal species that co-occur with *Coccidioides*, the most abundant of which is found commonly in rodent seed caches, implicating the current or past activity of rodents in *Coccidioides* detection. A caveat here, though, was our limited sequencing depth, which infrequently detected *Coccidioides*, and likely missed similarly rare species, which indicates that a higher sequencing depth investigation of the association between the fungal community and *Coccidioides* is needed. Finally, our low detection rate of *Coccidioides* in settled dust, combined with the difficulty other researchers have faced detecting *Coccidioides* in air, has led us to speculate that *Coccidioides* may not be well adapted to airborne transmission, instead relying primarily on inhalation by near-surface mammals. However, the low amounts of DNA we recovered from our settled dust samples, the fact that concentrating our DNA samples from settled dust allowed us to detect *Coccidioides* where we previously had been unable and the incidence of respiratory coccidioidomycosis in humans indicates that an expanded sampling effort and better DNA extraction methods are needed moving forward. With regard to rodent burrow soils, we recommend that potential animal hosts be investigated using immunoassays for *Coccidioides* antibodies [32] to better develop ecological models regarding *Coccidioides* and seasonality, and to better explain the temporal deviation we show between *Coccidioides* detection in soil and coccidioidomycosis incidence. We further suggest that the role burrowing rodents play in soil erosion be investigated, which may provide a better understanding of *Coccidioides* dispersal on undeveloped land. As we found strong site differences in *Coccidioides* detection on undeveloped lands, a missed opportunity here was a comparison of ecological landscape characteristics, such as vegetation cover, rodent burrow density and physical soil properties, which we recommend including in future studies. Finally, we suggest that large-scale air monitoring networks be leveraged for detection of *Coccidioides* in outdoor air and that that a better understanding of the CocciENV assay be pursued regarding low concentration samples from a variety of soil types and sampling mediums.

## Materials and methods

We investigated the presence of *Coccidioides* at nine sites, five on undeveloped land and four on agricultural land, which are situated in an area of nearly 10,000 km^2^ across three counties (Kern, Kings, and Fresno) in California’s San Joaquin Valley (SJV) (Fig 1). Soil was collected from within rodent burrows at the five undeveloped sites, which showed no evidence of recent cultivation or irrigation and were situated in a region known to harbor *Coccidioides* in soils [17,82,113]. These five undeveloped sites were all adjacent to California Highway 33 (Hwy33), have aridic soils, little annual precipitation, and wild vegetation composed primarily of *Nassella spp*., *Sporobolus spp*. and *Suaeda nigra* [114]. Soil was also collected at and below the surface of row crop, tree crop, and fallow plots at four agricultural sites in the SJV: the West Side Research and Extension Center (West Side) and the Kearney Agricultural Research and Extension Center (Kearney), both managed by the University of California division of Agriculture and Natural Resources; the Shafter Research Station (Shafter), managed by the San Joaquin Valley Quality Cotton Growers Association; and a private organic farm south of Bakersfield near Weedpatch, California (Weedpatch). No rodent burrow soils were collected from West Side, Shafter, Weedpatch or Kearney because we were unable to locate any rodent burrows either within or on the periphery of the agricultural land investigated, though this does not preclude their presence on adjacent private land. No rodent burrow samples were collected at Kearney as sampling at this site had been done as part of a previous study that did not focus on rodent burrows and because rodents are excluded or actively removed from the Kearney site [57,58,109]. Given the sporadic distribution of *Coccidioides* in soils [16], we opted for sampling at a greater number of sites, rather than investigating certain within-site variables (e.g. soil depth). As we were unable to detect *Coccidioides* in any of our 472 agricultural soils samples, we decided against additional sampling”.

### Sample collection and DNA extraction

We collected 238 rodent burrow soil samples from Hwy33 sites, which were obtained by inserting hemispherical collectors into rodent burrows as deeply as possible and no deeper than 30cm. Sampling was carried out monthly from November 2017 through October 2018 from at least 3 burrows at each Hwy33 site. More samples were collected in November 2017 (+40 burrows), after which sampling was reduced to three samples per month due to time constraints, with smaller numbers of additional samples taken in January (+1 burrow), May (+6 burrows), August (+6 burrows), and September (+6 burrows). The same burrows were not necessarily sampled each month. One burrow sample from site 2 in January was lost. At Kearny, 145 composite soil samples were collected from within sorghum fields during the summers of 2016 (May–September), 2017 (June–October) and 2018 (July–October) as part of a previous study [57,58]. Each composite soil sample collected at Kearney consisted of ten 15cm deep and 3cm diameter soil cores collected from each experimental plot that was then mixed using a glass stirring rod in a plastic vessel. Agricultural soils from Weedpatch, West Side and Shafter were collected from the soil surface, no deeper than 5cm, within the cultivated or cultivable portions of agricultural fields. We collected 132 soil samples from Weedpatch in April 2021, with 12 composite samples (composed of 11 individual soil samples each) collected as transects along the edge of fields cultivated with carrot, pepper and radish (2 transects each), and brassica, chard, cilantro, fennel, lettuce and parsley (1 transect each). Sampling at West Side took place in July 2021 and provided 143 samples from two fields that had been out of production for one year. In the first field, 13 individual samples were collected at the corners of 10, 20, 40 and 80m nested squares. In the second field, 10 composite samples were obtained, each one composed of 13 individual soil samples collected along 90m transects. Sampling at Shafter occurred in October 2021 and provided 52 samples, with 13 individual soil samples collected at the corners of 1, 2, 4 and 8m nested squares from one cotton field, one almond orchard, and two fallowed fields (one 6 years old and the other 12 years old). Except for Kearney samples, each soil sample was mixed by inverting the collection tube several times in the field, and DNA was extracted from all samples from a 0.25g soil subsample using the MoBio Powersoil DNA kit (MoBio, Carlsbad, CA, USA). DNA quantity was measured with the Qubit dsDNA HS Assay kit (Life Technologies Inc., Gaithersburg, MD, USA).

Airborne fungi were sampled from dust that settled passively into empty, sterile Petri dishes shielded from precipitation and perched atop polyvinylchloride pipes mounted on steel posts 50cm above the soil surface, as previously described [17,57,58]. Settled dust was collected monthly from all Hwy33 sites from November 2017 through October 2018 (175 samples) and, again monthly, from Kearney in September and October in 2017 and from May through September in 2018 (90 samples). Settled dust was recovered from Petri dishes by moistening sterile, DNA-free cotton swabs (Puritan, Guilford, ME, USA) in DNA-free distilled water and swabbing the inside surface. DNA extraction from the swab tips also used the MoBio Powersoil DNA kit (MoBio, Carlsbad, CA, USA), and DNA quantity was measured, again, with the Qubit dsDNA HS Assay kit (Life Technologies Inc., Gaithersburg, MD, USA).

### *Coccidioides* detection using the CocciEnv assay

*Coccidioides* from all soil and settled dust samples was detected using the CocciEnv qPCR assay, which uses 29 primer variants to target a repeating transposon sequence unique to the *Coccidioides* genome (NCBI BioProject PRJNA46299), representing up to 496 estimated unique alleles [84]. Reactions were run on 96 well plates at 20μl per reaction, each containing 2μl CocciEnv primer mix, which contains the 29 primers and Taqman probe (Applied Biosystems, Waltham, MA, USA) in the concentrations outlined in Bowers et al. (2019). Each reaction also contained 10μl of TaqMan Environmental Master Mix 2.0 (Applied Biosystems, Waltham, MA, USA), 6μl nuclease-free H_2_O and 2μl of template DNA. All DNA from soil samples was standardized to 50ng·μl^-1^ while DNA from settled dust samples not standardized due to low extracted DNA concentrations. The reaction was carried out on a Stratagene Mx3000P qPCR system (Agilent Technologies, Santa Clara, CA, USA) with an initial cycle at 95°C for 10 minutes, 40 cycles at 95°C for 15 seconds each and a final cycle at 60°C for 1 minute. Nuclease-free water was used as a negative control and DNA extracted from cultured *C*. *posadasii* strain Silveira was used as a positive control. Amplification curves were processed using Mxpro version 4.1 (Agilent Technologies, Santa Clara, CA, USA) with the relative fluorescence unit threshold set at 1000. An individual well was considered positive for *Coccidioides* at cycle threshold (CT) < 40, with logarithmic amplification, and when standards and controls behaved as expected. All soil samples were run in quadruplicate wells, replicated on two separate plates, for a total of eight reactions each. Due to low recovered DNA quantities (in many cases, <0.005 ng·μl^-1^), settled dust samples were initially run in triplicate wells on a single plate. Afterwards, the DNA from any settled dust sample with at least one positive well was concentrated in a vacuum oven (20kPa at approximately 65°C) for 1 hour or until the entire volume had evaporated and then resuspended in nuclease-free H_2_O. The CocciEnv assay was then run again using the entire concentrated DNA sample (S2 Table) in quadruplicate wells on a single plate. As qPCR amplification can produce false positives in the absence of template DNA [112], positive wells were summed to assign confidence on a qualitative scale. Samples were considered positive with ≥ 75% positive wells (≥ 6/8 wells for soil samples and ≥ 3/4 wells for settled dust samples). As there are no standard criteria for addressing ambiguity across replicates for either the CocciEnv or CocciDx assay, we opted to include as much information as possible (and all necessary data) such that the reader can draw their own conclusions. Our criterion for sample positivity was arbitrarily chosen, though it is conservative in that it allows that some samples that *may be positive* for *Coccidioides* are not labeled as negative. We have chosen this criterion because we prefer to err on the side of caution, and in this regard false positive results are preferable to false negative results. The fewer number of replicate plates (1 plate instead of 2) used for settled dust samples introduces uncertainty, though allows for a higher concentration of DNA per reaction. Importantly, as is the case with all such assays, the one performed here can only show that *Coccidioides* is present at a given location and cannot confirm that *Coccidioides* is absent, only that it is not detected. All qPCR data has been deposited in the Dryad repository (www.datadryad.org): https://doi.org/10.5061/dryad.p8cz8w9vx [115].

### ITS2 fungal community sequencing

In order to investigate the relationship between *Coccidioides* and the broader soil fungal community, we compared *Coccidioides* detection using the CocciEnv qPCR assay with the composition and structure of the fungal community using ITS2 sequencing data acquired from the same samples (and DNA extractions), as published in previous work [17,57,58]. We limited these analyses to only rodent burrow soil samples from Hwy33, being the only samples where *Coccidioides* was detected. The PCR amplification primers, 5.8SFun (AACTTTYRRCAAYGGATCWCT) and ITS4Fun (AGCCTCCGCTTATTGATATGCTTAART) [116] were used to amplify the internal transcribed spacer 2 (ITS2) region of fungal ribosomal DNA. The reaction mixture used contained 2.5μl of 50μM forward and reverse primers, 2μl of template DNA 2.5μl of nuclease-free water, 3μl of BSA and 12.5μl of AccuStart II PCR SuperMix kit (Quantabio, Beverly, MA, USA). The Gene Amplification PCR System (Bio-Rad Laboratories, Hercules, CA, USA) was used to perform that PCR reaction with 1 cycle of 96°C for 2 minutes, 35 cycles of 94°C for 30 seconds, 58°C for 40 seconds and 72°C for 2 minutes, and 1 cycle of 72°C for 10 minutes. The protocol we used for PCR amplification of soil and settled dust samples from Kearney sites was identical to that for Hwy33 sites with these differences: Template DNA was diluted to 5ng⸱ μl^-1^, 5PRIME HotMaster Mix (Eppendorf-5Prime, Gaithersburg, MD, USA) was used in the place of AccuStart II PCR SuperMix, and no BSA was added to the PCR reaction. These differences were due to the discontinuation of the AccuStart II PCR SuperMix kit. The PCR product was then sequenced at the QB3 Vincent J. Coates Genomics Sequencing Laboratory (University of California, Berkeley, CA, USA), following quantification using the Qubit dsDNA HS Assay kit (Life Technologies Inc., Gaithersburg, MD, USA). Sequencing was performed on the Illumina MiSeq with the PE300 paired-end chemistry (Illumina, Inc., CA, USA) using unique dual indices.

### Bioinformatics

Raw sequence files were processed in Qiime 2 version 2019.10.0 [117]. Sequencing runs were manually inspected followed by denoising in DADA2 [118]. Next, paired-end reads were joined, end bases below a quality score of 25 were removed and unpaired reads were discarded. Operational taxonomic units (OTUs) were assigned to taxonomic groups using a naïve Bayes classifier trained at 97% similarity with the UNITE database [119–121]. OTUs that were matched to an a UNITE database entry that has not been identified were labeled as “Unidentified”. OTUs that were algorithmically assigned to a specific taxonomic level but could not be matched to a UNITE database entry were label as “Unspecified”. All sequence data supporting the findings of this study and accompanying metadata have been deposited in the NCBI Sequence Read Archive (www.ncbi.nlm.nih.gov/sra) with the following accession numbers: PRJNA736543 [122], PRJNA736167 [123] and PRJNA736519 [124]. All code used to create taxonomic tables from raw sequencing data has been previously published [17].

### Environmental data

Realizing that time embraces many temporally explicit variables (e.g., seasonal variation in temperature, moisture, vegetation, etc.), environmental, remotely sensed variables were investigated and included in statistical models. Normalized Difference Vegetation Index (NDVI) and Enhanced Vegetation Index (EVI) data were acquired from the MODIS/Terra Vegetation Index dataset at a 16day, 250m resolution [125]. Soil moisture data was acquired from the L3 radar/radiometer global EASE-grid soil moisture dataset version 3 at a daily 9km resolution [126]. Estimated minimum temperature, maximum temperature and total precipitation was acquired from the Daymet model (Oak Ridge National Laboratory, Tennessee, USA) at a daily resolution [127]. All remote sensing satellite data was acquired through the NASA AppEEARS data portal version 3.3.1 [128] (https://appeears.earthdatacloud.nasa.gov) and has been summarized (S1 Fig) and deposited in the Dryad repository (www.datadryad.org): https://doi.org/10.5061/dryad.p8cz8w9vx [115]. Remote sensing data was extracted for Hwy33 sites using the “point sample tool” in the AppEEARS data portal and monthly mean values were calculated. Unfortunately, no laboratory analytical chemistry or physical analyses were done on soil samples due to biosafety constraints.

### Statistical analysis

Statistical analyses used the R programming language version 4.1.0 [129] and vegan version 2.5.7 [130]. Significant differences in the detection of *Coccidioides* in soils between sites (categorical) and months (categorical) were determined using an additive only (no interactions) logistic regression model with the “glm” function using a binomial distribution. Logistic regression with a binomial distribution was used (as opposed to, for example, OLS regression) because our independent variable, *Coccidioides* detection, is binary (present or absent). As it has been hypothesized that *Coccidioides* growth may be corelated with soil moisture [18], additional remotely-sensed data, inferring or covarying with soil moisture (temperature maximum, temperature minimum, precipitation, SMAP-derived soil moisture, NDVI and EVI), were included in the model as continuous variables and the model was re-run. Overall model fit for logistic regression was calculated as McFadden’s pseudo r^2^ “rho squared” (ρ^2^) (with accepted good fits from 0.20–0.40) [131] because r^2^ cannot be calculated for logistic regression in the same manner as OLS regression [132]. Significance of the full logistic regression model was determined from the predictor degrees of freedom and the Chi-square statistic (which was obtained by subtracting the residual deviance from the null deviance of the model). To compare *Coccidioides* detection between different factor levels, pairwise differences between sites and months were calculated from t-ratios derived from the predicted estimated marginal mean difference between each factor combination [133] using the “emmeans” package version 1.7.1.1 [134], and p-values were Tukey-adjusted. Coccidioides detection in settled dust was not analyzed statistically due to the low number of positive samples (5 / 265).

Analysis of fungal community sequence data was done at the species level. Initially, species with only a single instance across all samples were removed. Species counts were then square-rooted and Wisconsin double standardized (each taxon was divided by the most abundant taxon among all samples, and then divided across all taxa within each sample to calculate a proportional relative effect size). This standardization procedure reduces the influence of abundant taxa and facilitates comparisons across samples of different sequencing depths. Finally, fungal community β-diversity was calculated as Bray-Curtis dissimilarity [135,136]. Comparing community β-diversity and environmental gradients is widely used in community ecology to identify patterns and gain insights into concepts ranging from climate change [137,138] to soil ecology [139] and airborne fungal pathogen dispersal [140]. A fully factorial permutational multivariate analysis of variance (PERMANOVA) test was used to determine whether fungal community β-diversity was correlated with the presence of *Coccidioides*, spatial-temporal differences as categorical variables (site and month) or remotely sensed data as continuous variables (temperature maximum, temperature minimum, precipitation, soil moisture, NDVI and EVI) using the “adonis2” function with 1000 permutations [141]. PERMANOVA tests are used commonly in community ecology and were used here instead of traditional multivariate analysis of variance tests because of the advantages of a non-parametric approach, especially with regard to non-normally distributed, zero-inflated microbial species count data. In order to try and identify fungal species that may co-occur with *Coccidioides*, an analysis of indicator species was performed on untransformed species data from Hwy33 rodent burrow soil samples using the IndVal metric with indicspecies version 1.7.12 [142,143]. Sequences associated with taxa identified as possible indicator species (p < 0.05, reads ≥ 100) were reanalyzed using BLASTN (https://blast.ncbi.nlm.nih.gov) and likely species identities (> 96% identity) were cross-referenced with species names in the Mycobank database (https://www.mycobank.org). In all statistical tests, sampling month was treated as a categorical variable because more advanced non-linear models (harmonic or spline regression) performed less well than our categorical model. To compare categorical, harmonic regression and spline regression model performance, a second-order (due to high model parameter to sample size ratios) Akaike Information Criterion (AICc) was used [144], and models with spline or harmonic terms were compared with null models lacking such terms using a likelihood ratio test. Visualizations were created using ggplot2 version 3.3.5 [145] and Complexheatmap version 2.8.0 [146]. Mapping used osmdata version 0.1.8 [147] and ggmap version 3.0.0 [148], with map tiles by Stamen Design (www.stamen.com) under Creative Commons By Attribution (CC BY 3.0) and map data by OpenStreetMap (www.openstreetmap.org) under the Open Data Commons Open Database License (ODbL). California shapefile is public domain data by Natural Earth (www.naturalearthdata.com).

## Dryad DOI

10.5061/dryad.p8cz8w9vx

## Supporting information

S1 Fig Remote sensing satellite data for Hwy33 sites. (DOCX)

S2 Fig The predicted probability of detecting *Coccidioides* with remotely sensed data. (DOCX)

S3 Fig The predicted probability of detecting *Coccidioides* using harmonic regression and spline regression. (DOCX)

S4 Fig PCoA plot for the top 10 and top 50 most abundant species. (DOCX)

S5 Fig The 50 most abundant fungal species in rodent burrow soils. (DOCX)

S6 Fig The 50 most abundant fungal species in settled dust samples. (DOCX)

S1 Table Sampling enumeration for the current study and other studies where environmental *Coccidioides* was detected. (DOCX)

S2 Table DNA concentration in settled dust samples. (DOCX)

S3 Table Logistic regression coefficient table with remote sensing data. (DOCX)

S4 Table Pairwise differences between sites and months. (DOCX)

S5 Table Comparison between models using harmonic, spline or categorical terms for sampling month. (DOCX)

S6 Table PERMANOVA coefficient table with remote sensing data. (DOCX)

S7 Table Top 10 species removed PERMANOVA coefficient table. (DOCX)

S8 Table Top 50 species removed PERMANOVA coefficient table. (DOCX)

S9 Table Top 10 species removed PERMANOVA coefficient table with remote sensing data. (DOCX)

S10 Table Top 50 species removed PERMANOVA coefficient table with remote sensing data. (DOCX)

S11 Table Consolidated indicator species for *Coccidioides* positive and *Coccidioides* negative rodent burrow soil samples. (DOCX)
