# Supplementary material for: Coccidioides undetected in soils from agricultural land and uncorrelated with time or the greater soil fungal community on undeveloped land
Source: PLoS Pathog. 2023 May 25;19(5):e1011391. doi: 10.1371/journal.ppat.1011391 (PMC10246812; doi:10.1371/journal.ppat.1011391)
Supplement: S1 Fig — (DOCX) [file ppat.1011391.s001.docx]

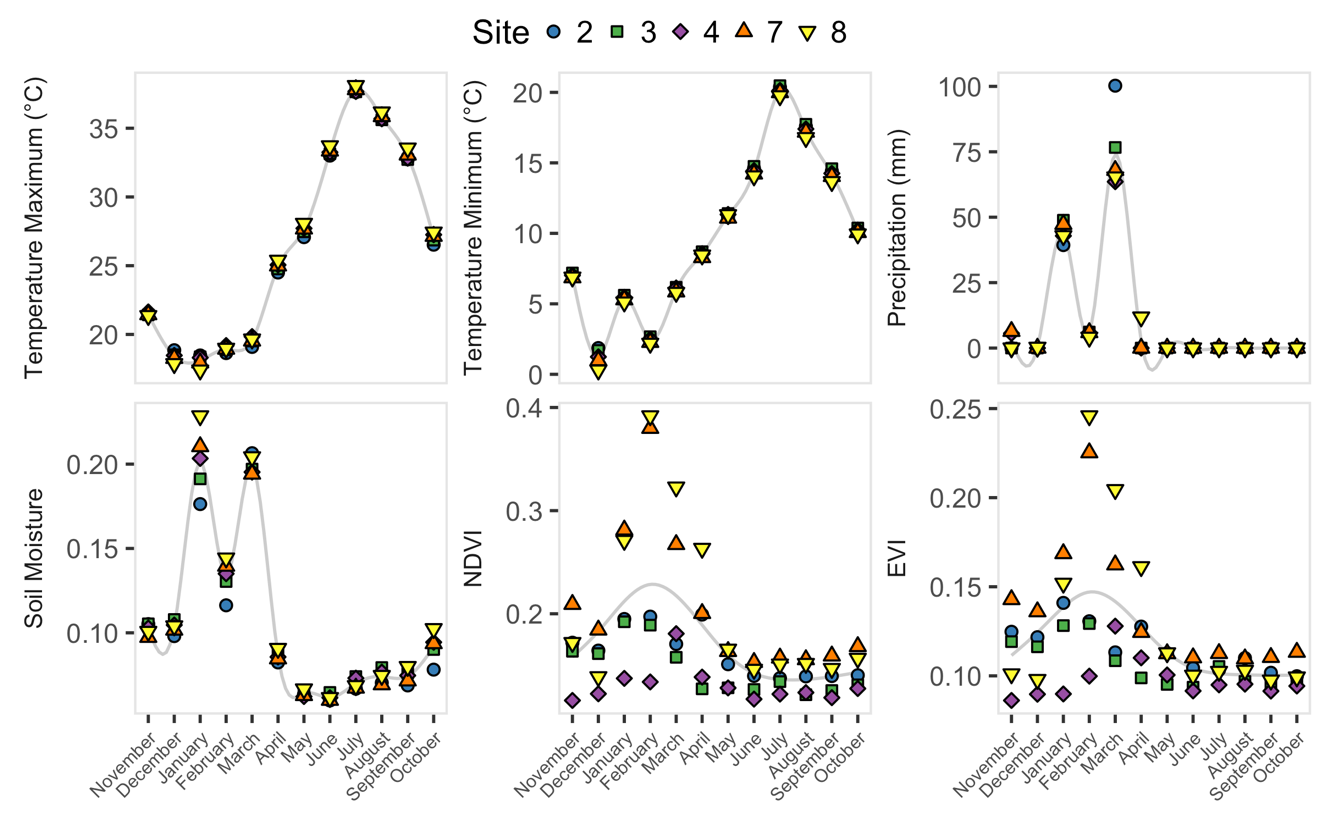


**Figure S1.** Remote sensing satellite data for Hwy33 sites acquired through the NASA AppEEARS data portal. Temperature data = monthly mean. Precipitation data = monthly total. Soil moisture data = monthly mean (cm^3^/cm^3^). Gray line connects mean monthly values using a general additive model (12 maximum degrees of freedom). n = 60.
