# Supplementary material for: Coccidioides undetected in soils from agricultural land and uncorrelated with time or the greater soil fungal community on undeveloped land
Source: PLoS Pathog. 2023 May 25;19(5):e1011391. doi: 10.1371/journal.ppat.1011391 (PMC10246812; doi:10.1371/journal.ppat.1011391)
Supplement: S2 Fig — (DOCX) [file ppat.1011391.s002.docx]

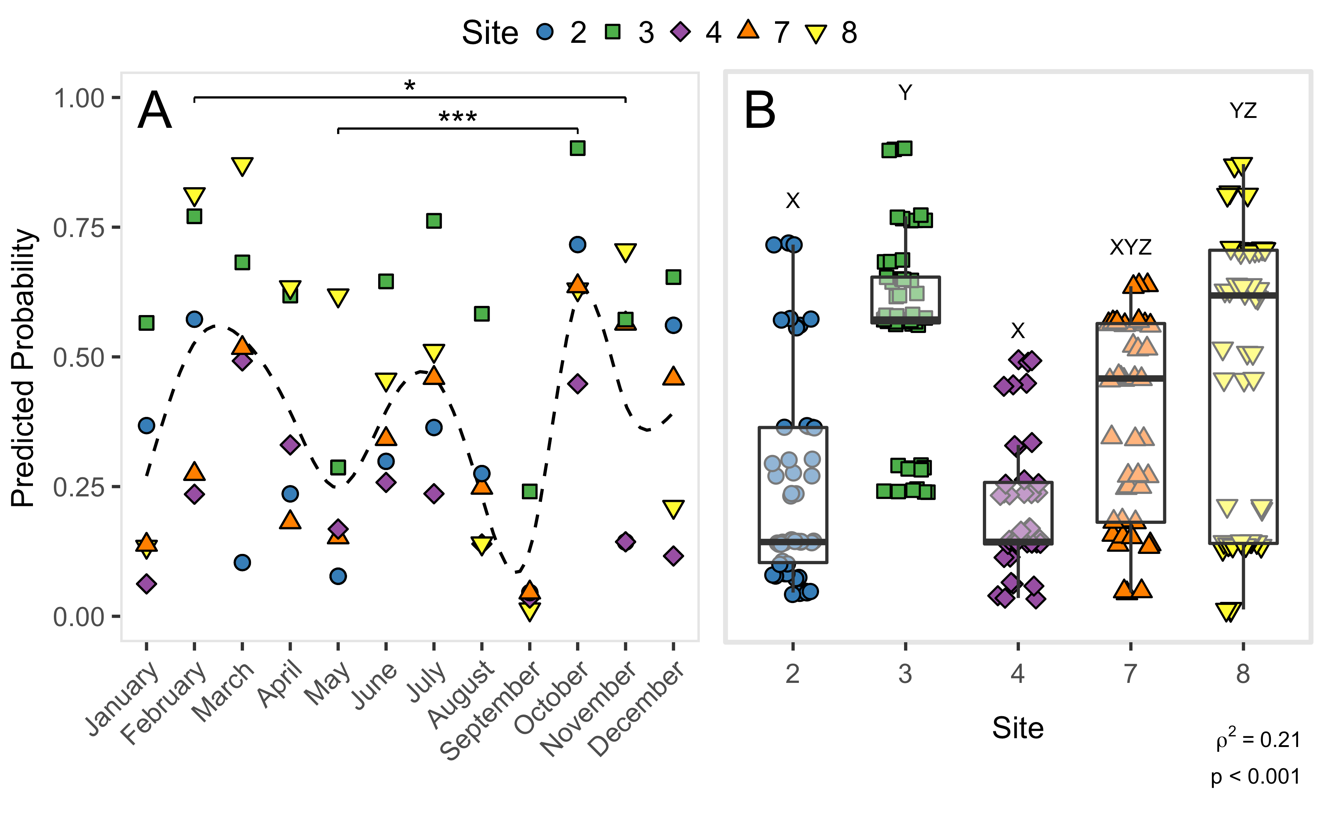


**Figure S2.** The predicted probability (derived from logistic regression) of detecting *Coccidioides* (as a function of month, site, and remotely sensed data (Temperature Maximum, Temperature Minimum, Precipitation, Soil Moisture, NDVI and EVI). Predicted probabilities are displayed as means as a function of month (A) and as individual samples as a function of site (B) (points jittered for clarity). * = months differ significantly (* = p < 0.05, ** = p < 0.01, *** = p ≤ 0.001, Tukey-adjusted pairwise contrasts). Letters (XYZ) denote groups of sites that do not significantly differ (p > 0.05, Tukey-adjusted pairwise contrasts). Total month contrasts = 66. Total site contrasts = 10. “rho squared” (ρ^2^) = McFadden's pseudo r^2^. p = significance of full logistic regression model derived from deviance and null deviance. n = 238. Dashed line connects monthly mean predicted probabilities (generalized additive model).
