# Supplementary material for: Coccidioides undetected in soils from agricultural land and uncorrelated with time or the greater soil fungal community on undeveloped land
Source: PLoS Pathog. 2023 May 25;19(5):e1011391. doi: 10.1371/journal.ppat.1011391 (PMC10246812; doi:10.1371/journal.ppat.1011391)
Supplement: S3 Fig — (DOCX) [file ppat.1011391.s003.docx]

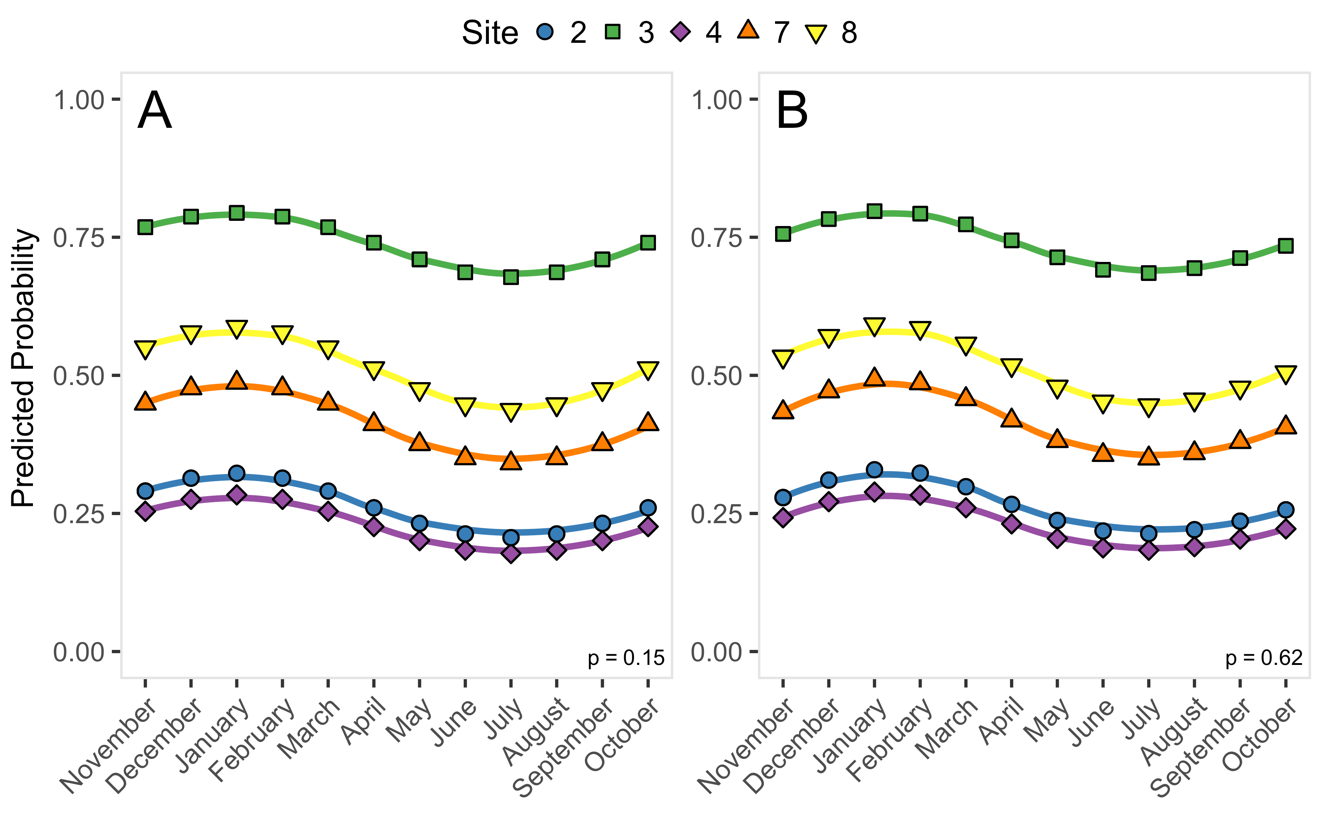


**Figure S3.** The predicted probability (derived from logistic regression) of detecting *Coccidioides* as a function of site and month using harmonic regression (A) and spline regression (B). p = significance (p-value) derived from a likelihood ratio test with a null model lacking the harmonic or spline month terms.
