# Supplementary material for: Coccidioides undetected in soils from agricultural land and uncorrelated with time or the greater soil fungal community on undeveloped land
Source: PLoS Pathog. 2023 May 25;19(5):e1011391. doi: 10.1371/journal.ppat.1011391 (PMC10246812; doi:10.1371/journal.ppat.1011391)
Supplement: S4 Fig — (DOCX) [file ppat.1011391.s004.docx]

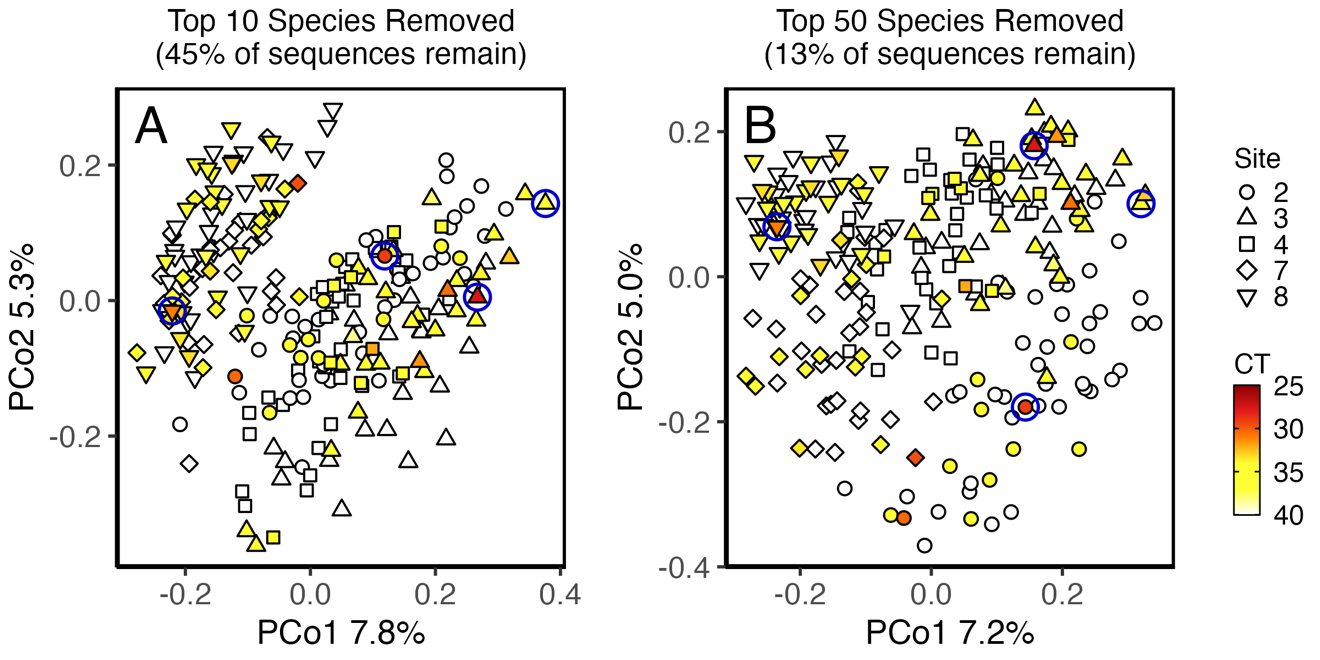


**Figure S4.** PCoA plot showing the Bray-Curtis dissimilarity within the fungal community between individual rodent burrow soil samples from Hwy33 sites with the top 10 (A) and top 50 (B) most abundant species removed from analysis. Colored points = samples positive for *Coccidioides* using the CocciENV qPCR assay. White points = samples negative for *Coccidioides* using the CocciENV qPCR assay (less than 6 positive wells – “Weak Positive”, “Low Replication”, “Unreplicated and “Undetected” samples). Blue circles = samples positive for *Coccidioides* in the ITS2 rDNA dataset. CT = qPCR cycle threshold value. n = 238.
