# Supplementary material for: Coccidioides undetected in soils from agricultural land and uncorrelated with time or the greater soil fungal community on undeveloped land
Source: PLoS Pathog. 2023 May 25;19(5):e1011391. doi: 10.1371/journal.ppat.1011391 (PMC10246812; doi:10.1371/journal.ppat.1011391)
Supplement: S5 Fig — (DOCX) [file ppat.1011391.s005.docx]

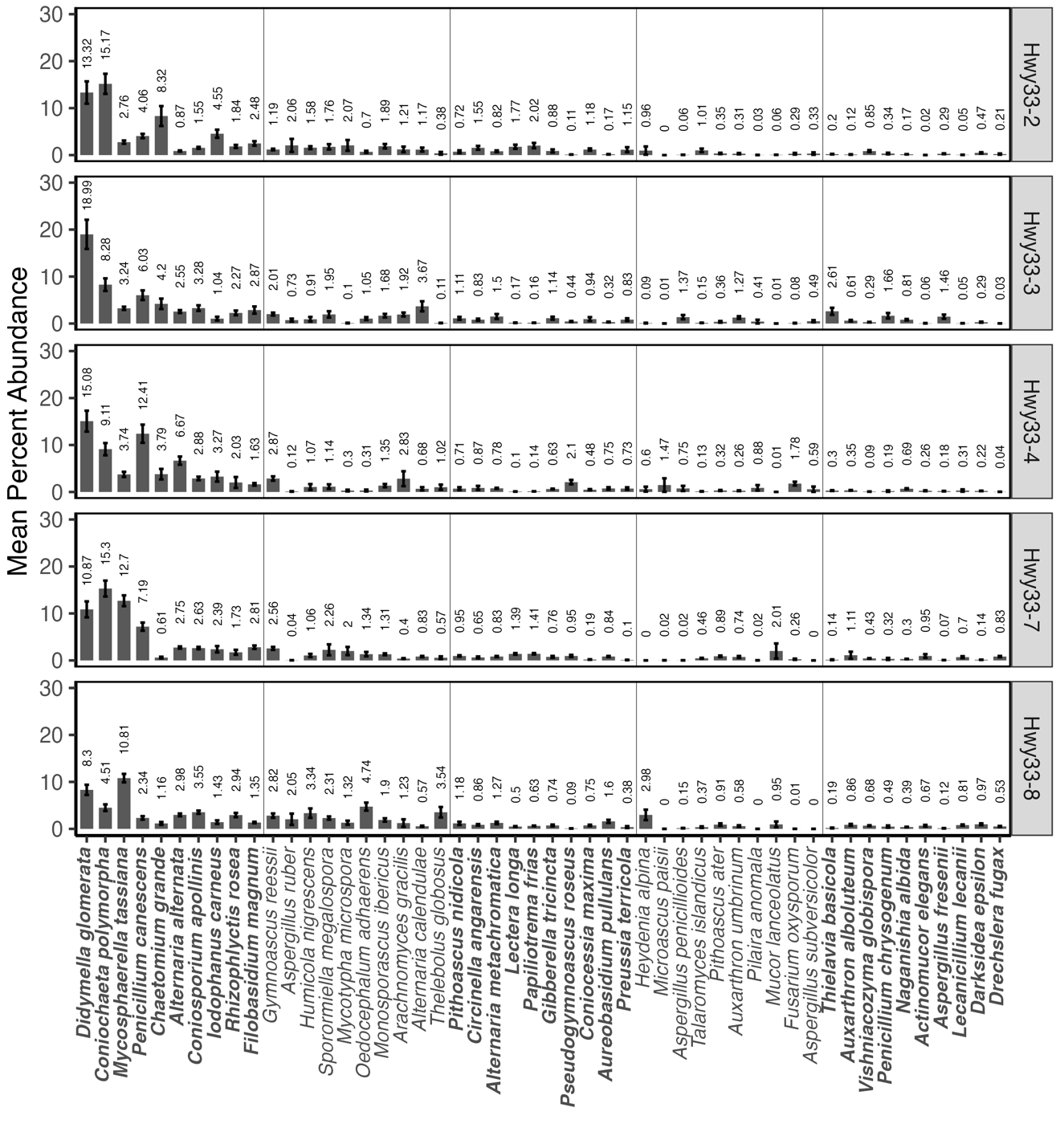


**Figure S5.** Mean percent abundance of the 50 most abundant fungal species as a function of sampling site in rodent burrow soils from Hwy33. n = 238. Error bars = SEM. Values < 0.005 were rounded down to 0.
