# Supplementary material for: Coccidioides undetected in soils from agricultural land and uncorrelated with time or the greater soil fungal community on undeveloped land
Source: PLoS Pathog. 2023 May 25;19(5):e1011391. doi: 10.1371/journal.ppat.1011391 (PMC10246812; doi:10.1371/journal.ppat.1011391)
Supplement: S6 Fig — (DOCX) [file ppat.1011391.s006.docx]

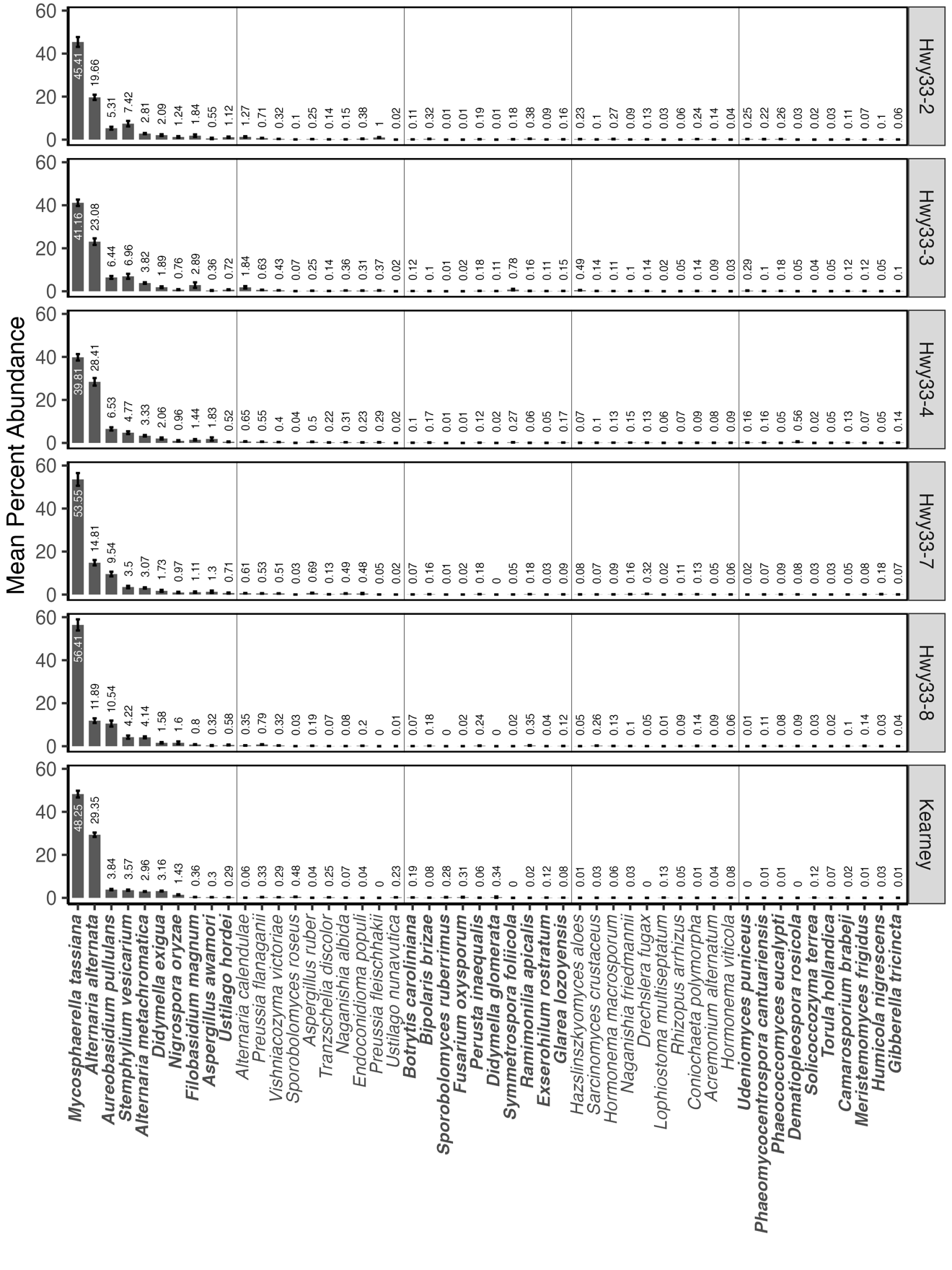


**Figure S6.** Mean percent abundance of the 50 most abundant fungal species as a function of sampling site in settled dust samples from Hwy33 and Kearney. n = 265. Error bars = SEM. Values < 0.005 were rounded down to 0.
