# Supplementary material for: Coccidioides undetected in soils from agricultural land and uncorrelated with time or the greater soil fungal community on undeveloped land
Source: PLoS Pathog. 2023 May 25;19(5):e1011391. doi: 10.1371/journal.ppat.1011391 (PMC10246812; doi:10.1371/journal.ppat.1011391)
Supplement: S1 Table — (DOCX) [file ppat.1011391.s007.docx]

**Table S1.** Sampling enumeration for the current study (yellow, bold text) and other studies where environmental *Coccidioides* was detected in soil, air, or both. Only one other study investigated *Coccidioides* in agricultural soils (green), while 16 studies investigated *Coccidioides* in non-agricultural soils. One study detected *Coccidioides* in both soils and in air (pink), and three studies detected *Coccidioides* only in air (blue). Total studies = 24. (+) = positive samples.

|  | Collection Location | Detection Method | Total | (+) Total | Burrow | (+) Burrow | non-Burrow | (+) non-Burrow | Agricultural | (+) Agricultural | Airborne | (+) Airborne |
| --- | --- | --- | --- | --- | --- | --- | --- | --- | --- | --- | --- | --- |
| **Current Study** | **California** | **CocciEnv qPCR** | **710** | **94** | **238** | **89** | **472** | **0** | **472** | **0** | **265** | **5** |
| Valley Fever: Environmental Risk Factors and Exposure Pathways Deduced from Field Measurements in California (Lauer et al. 2020) [1] | California | Nested PCR | 389 | 122 | 0 | 0 | 389 | 122 | 30-36 | 0 | 0 | 0 |
| Investigating the Role of Animal Burrows on the Ecology and Distribution of *Coccidioides* spp. in Arizona Soils (Kollath et al. 2020) [2] | Arizona | CocciDX/Env qPCR | 465 | 105 | 385 | 95 | 77 | 10 | 0 | 0 | 0 | 0 |
| Direct detection of *Coccidioides* from Arizona soils using CocciENV, a highly sensitive and specific real-time PCR assay (Bowers et al. 2019) [3] | Arizona | CocciEnv qPCR | 76 | 4 | 48 | 4 | 30 | 0 | 0 | 0 | 0 | 0 |
| Detection of *Coccidioides posadasii* from xerophytic environments in Venezuela reveals risk of naturally acquired coccidioidomycosis infections (Alvarado et al. 2018) [4] | Venezuela | CocciEnv qPCR | 15 | 9-15 | 0 | 0 | 15 | 9-15 | 0 | 0 | 0 | 0 |
| Demonstration of *Coccidioides* *immitis* and *Coccidioides* *posadasii* DNA in soil samples collected from Dinosaur National Monument, Utah  (Johnson et al. 2014) [5] | Utah | Nested PCR |  | 2 | 1 | 1 | 1 | 1 | 0 | 0 | 0 | 0 |
| *Coccidioides* *immitis* identified in soil outside of its known range - Washington, 2013 (Marsden-Haug et al. 2013) [6] | Washington | CocciDX qPCR | 22 | 6 |  |  |  |  | 0 | 0 | 0 | 0 |
| Detection and phylogenetic analysis of *Coccidioides posadasii* in Arizona soil samples (Barker et al. 2012) [7] | Arizona | PCR, Mouse, Culture | 700 | 11 |  | 5 |  | 6 | 0 | 0 | 0 | 0 |
| Detection of *Coccidioides immitis* in Kern County, California, by multiplex PCR (Lauer et al. 2012) [8] | California | PCR | 546 | 31 | 0 | 0 | 546 | 31 | 0 | 0 | 0 | 0 |
| Molecular identification of *Coccidioides* spp. in soil samples from Brazil (Macêdo et al. 2011) [9] | Brazil | Nested PCR, Mouse | 24 | 6 | 24 | 6 | 0 | 0 | 0 | 0 | 0 | 0 |
| Soil isolation and molecular identification of *Coccidioides* *immitis*  (Greene et al. 2000) [10] | California | PCR | 720 | 4 | 0 | 0 | 720 | 4 | 0 | 0 | 0 | 0 |
| Soil Ecology of *Coccidioides immitis* at Amerindian Middens in California (Lacy and Swatek 1974) [11] | California | Culture | 325 | 32 | 0 | 0 | 325 | 32 | 0 | 0 | 0 | 0 |
| Some fungi isolated with *Coccidioides immitis* from soils of endemic areas in California (Orr 1968) [12] | California | Culture | 22 | 22 | 0 | 0 | 22 | 22 | 0 | 0 | 0 | 0 |
| Observations on *Coccidioides immitis* found growing naturally in soil.  (Maddy et al. 1965) [13] | Arizona | Culture, Injection | 198 | 30 | 0 | 0 | 198 | 30 | 0 | 0 | 0 | 0 |
| Isolation of *Coccidioides immitis* from Soil (Levine et al. 1964) [14] | California | Culture, Mouse Injection | 37 | 4 |  |  |  |  | 0 | 0 | 0 | 0 |
| Growth Patterns of *Coccidioides immitis* (Elconin 1957) [15] | California | Culture, Mouse | 428 | 31 | 115 | 18 | 313 | 13 | 0 | 0 | 0 | 0 |
| Ecological studies of *Coccidioides immitis* (Plunkett and Swatek 1957) [16] | California | Culture | 80 | 0 | 80 | 0 | 0 | 0 | 0 | 0 | 0 | 0 |
| *Coccidioides immitis* in the soil of the southern San Joaquin Valley.  (Egeberg and Ely 1956) [17] | California | Culture, Injection | 500 | 35 | 177 | 24 | 323 | 11 | 0 | 0 | 0 | 0 |
| Isolation of *Coccidioides* from soil and rodents (Emmons 1942) [18] | Arizona | Isolation | 150 | 5 |  |  |  |  | 0 | 0 | 0 | 0 |
| An Epidemic Of Coccidioidal Infection (Coccidioidomycosis)  (Davis et al. 1942) [19] | California | Isolation | 1 | 1 | 1 | 0 | 0 | 0 | 0 | 0 | 0 | 0 |
| Isolation of *Coccidioides Immitis* (Stiles) from the Soil  (Stewart and Meyer 1932) [20] | California | Culture, Guinea Pig | >1 | >1 |  |  |  |  |  |  | 0 | 0 |
| Molecular detection of airborne *Coccidioides* in Tucson, Arizona  (Chow et al. 2016) [21] | Arizona | Single Tube Nested qPCR |  | 10 |  | 2 |  | 8 | 0 | 0 | 25 | 3 |
| The detection of *Coccidioides* from ambient air in Phoenix, Arizona: Evidence of uneven distribution and seasonality (Gade et al. 2020) [22] | Arizona | Single Tube Nested qPCR | 0 | 0 | 0 | 0 | 0 | 0 | 0 | 0 | 1009 | 96-135 |
| Development of a Quantitative TaqMan-PCR Assay and Feasibility of Atmospheric Collection for *Coccidioides immitis* for Ecological Studies (Daniels et al. 2002) [23] | California | PCR | 0 | 0 | 0 | 0 | 0 | 0 | 0 | 0 | 12 | 4 |
| Recovery of *Coccidioides immitis* from the air (Ajello et al. 1965) [24] | Arizona | Mouse Injection, Culture | 0 | 0 | 0 | 0 | 0 | 0 | 0 | 0 | 128 | 2 |

**References**

1. Lauer A, Etyemezian V, Nikolich G, Kloock C, Arzate AF, Sadiq Batcha F, et al. Valley fever: Environmental Risk Factors and Exposure Pathways Deduced from Field Measurements in California. IJERPH. 2020;17: 5285. doi:10.3390/ijerph17155285

2. Kollath DR, Teixeira MM, Funke A, Miller KJ, Barker BM. Investigating the role of animal burrows on the ecology and distribution of *Coccidioides* spp. in Arizona soils. Mycopathologia. 2020;185: 145–159.

3. Bowers J, Parise K, Kelley E, Lemmer D, Schupp J, Driebe E, et al. Direct detection of *Coccidioides* from Arizona soils using CocciENV, a highly sensitive and specific real-time PCR assay. Medical mycology. 2019;57: 246–255.

4. Alvarado P, Teixeira M de M, Andrews L, Fernandez A, Santander G, Doyle A, et al. Detection of *Coccidioides posadasii* from xerophytic environments in Venezuela reveals risk of naturally acquired coccidioidomycosis infections. Emerging microbes & infections. 2018;7: 1–13.

5. Johnson SM, Carlson EL, Fisher FS, Pappagianis D. Demonstration of *Coccidioides immitis* and *Coccidioides posadasii* DNA in soil samples collected from Dinosaur National Monument, Utah. Sabouraudia. 2014;52: 610–617.

6. Marsden-Haug N, Hill H, Litvintseva AP, Engelthaler DM, Driebe EM, Roe CC, et al. *Coccidioides immitis* identified in soil outside of its known range—Washington, 2013. MMWR Morbidity and mortality weekly report. 2014;63: 450.

7. Barker BM, Tabor JA, Shubitz LF, Perrill R, Orbach MJ. Detection and phylogenetic analysis of *Coccidioides posadasii* in Arizona soil samples. Fungal Ecology. 2012;5: 163–176. doi:10.1016/j.funeco.2011.07.010

8. Lauer A, Baal JDH, Baal JCH, Verma M, Chen JM. Detection of *Coccidioides immitis* in Kern County, California, by multiplex PCR. Mycologia. 2012;104: 62–69.

9. De Macêdo RC, Rosado AS, Da Mota FF, Cavalcante MA, Eulálio KD, Martins L, et al. Molecular identification of Coccidioides spp. in soil samples from Brazil. BMC microbiology. 2011;11: 1–9.

10. Greene DR, Koenig G, Fisher MC, Taylor JW. Soil isolation and molecular identification of *Coccidioides immitis*. Mycologia. 2000;92: 406–410.

11. Lacy GH, Swatek FE. Soil ecology of *Coccidioides immitis* at Amerindian middens in California. Applied microbiology. 1974;27: 379–388.

12. Orr G. Some fungi isolated with *Coccidioides immitis* from soils of endemic areas in California. Bulletin of the Torrey Botanical Club. 1968; 424–431.

13. Maddy K. Observations on *Coccidioides immitis* found growing naturally in soil. Arizona Med. 1965;22: 281–288.

14. Levine H, Winn WA, others. Isolation of *Coccidioides immitis* from Soil. Health laboratory science. 1964;1: 29–32.

15. Elconin AF, Egeberg M, Lubarsky R. Growth Patterns of *Coccidioides immitis*. Proceedings of Symposium on Coccidioidomycosis: Held at Phoenix, Ariz-Feb 11-13, 1957. US Department of Health, Education, and Welfare Public Health Service …; 1957. p. 168.

16. Plunkett O, Swatek F. Ecological studies of *Coccidioides immitis*. Proceedings of the Symposium on Coccidioidomycosis. AZ. Public Health Service Washington DC.; 1957. pp. 158–160.

17. Egeberg R, Ely AF. *Coccidioides immitis* in the soil of the southern San Joaquin Valley. American Journal of Medical Sciences. 1956;231: 151–4.

18. Emmons CW. Isolation of *Coccidioides* from soil and rodents. Public Health Reports (1896-1970). 1942;57: 109–111.

19. Davis BL, Smith RT, Smith CE. An epidemic of coccidioidal infection (coccidioidomycosis). Journal of the American Medical Association. 1942;118: 1182–1186.

20. Stewart R, Meyer K. Isolation of *Coccidioides immitis* (Stiles) from the soil. Proceedings of the Society for Experimental Biology and Medicine. 1932;29: 937–938.

21. Chow NA, Griffin DW, Barker BM, Loparev VN, Litvintseva AP. Molecular detection of airborne *Coccidioides* in Tucson, Arizona. Medical mycology. 2016;54: 584–592.

22. Gade L, McCotter OZ, Bowers JR, Waddell V, Brady S, Carvajal JA, et al. The detection of *Coccidioides* from ambient air in Phoenix, Arizona: Evidence of uneven distribution and seasonality. Medical Mycology. 2020;58: 552–559. doi:10.1093/mmy/myz093

23. Daniels J, Wilson W, DeSantis T, Gouveia F, Anderson G, Shinn J, et al. Development of a Quantitative TaqMan-PCR Assay and Feasibility of Atmosphoric Collection for *Coccidioides Immits* for Ecological Studies. Lawrence Livermore National Lab., CA (US); 2002.

24. Ajello L, Maddy K, Crecelius G, Hugenholtz PG, Hall LB. Recovery of *Coccidioides immitis* from the air. Med Mycol. 1965;4: 92–95. doi:10.1080/00362176685190231
