# Supplementary material for: Coccidioides undetected in soils from agricultural land and uncorrelated with time or the greater soil fungal community on undeveloped land
Source: PLoS Pathog. 2023 May 25;19(5):e1011391. doi: 10.1371/journal.ppat.1011391 (PMC10246812; doi:10.1371/journal.ppat.1011391)
Supplement: S2 Table — (DOCX) [file ppat.1011391.s008.docx]

**Table S2.** Settled dust samples initially showing *Coccidioides* detection in ≥1 well. Initial DNA concentration and DNA concentration after concentrating is shown as well as *Coccidioides* detection in the confirmation assay.

| Sample | Site | Year | Month | Day | Initial DNA (ng·µl^-1^) | Concentrated DNA (ng·µl^-1^) | Positive Wells | Detection |
| --- | --- | --- | --- | --- | --- | --- | --- | --- |
| Y171011Hwy2b | Hwy33 2 | 2017 | October | 11 | <0.005 | <0.005 | 0/4 | Negative |
| Y171011Hwy7b | Hwy33 7 | 2017 | October | 11 | <0.005 | 0.062 | 0/4 | Negative |
| Y171108Hwy7a | Hwy33 7 | 2017 | November | 8 | <0.005 | 0.11 | 0/4 | Negative |
| Y180118Hwy3a | Hwy33 3 | 2018 | January | 18 | <0.005 | <0.005 | 0/4 | Negative |
| Y180118Hwy4a | Hwy33 4 | 2018 | January | 18 | <0.005 | 0.146 | 2/4 | Negative |
| Y180118Hwy8a | Hwy33 8 | 2018 | January | 18 | <0.005 | 0.07 | 4/4 | Positive |
| Y180215Hwy3a | Hwy33 3 | 2018 | February | 15 | <0.005 | 0.13 | 4/4 | Positive |
| Y180215Hwy8a | Hwy33 8 | 2018 | February | 15 | <0.005 | 0.59 | 4/4 | Positive |
| Y180317Hwy2a | Hwy33 2 | 2018 | March | 17 | <0.005 | 0.157 | 0/4 | Negative |
| Y180317Hwy3a | Hwy33 3 | 2018 | March | 17 | 0.166 | 0.512 | 1/4 | Negative |
| Y180317Hwy4a | Hwy33 4 | 2018 | March | 17 | 0.074 | 0.117 | 1/4 | Negative |
| Y180317Hwy7a | Hwy33 7 | 2018 | March | 17 | <0.005 | 0.287 | 1/4 | Negative |
| Y181016Hwy8a | Hwy33 8 | 2018 | October | 16 | <0.005 | 0.364 | 3/4 | Positive |
| Y18A80726 | Kearney | 2018 | July | 26 | <0.005 | 0.155 | 4/4 | Positive |
| Y18B80925 | Kearney | 2018 | September | 25 | 0.692 | 0.467 | 0/4 | Negative |
