# Supplementary material for: Coccidioides undetected in soils from agricultural land and uncorrelated with time or the greater soil fungal community on undeveloped land
Source: PLoS Pathog. 2023 May 25;19(5):e1011391. doi: 10.1371/journal.ppat.1011391 (PMC10246812; doi:10.1371/journal.ppat.1011391)
Supplement: S3 Table — (DOCX) [file ppat.1011391.s009.docx]

**Table S3.** Logistic regression coefficient table (using the “glm” function) showing *Coccidioides* detection, using the CocciEnv qPCR assay, as a function of sampling site, sampling month and remotely sensed data. n = 238.

|  | Estimate | Standard Error | z-value | p-value |  |
| --- | --- | --- | --- | --- | --- |
| Intercept | 18.925 | 16.525 | 1.145 | 0.252 |  |
| February | 8.446 | 4.901 | 1.723 | 0.085 | . |
| March | 0.521 | 1.821 | 0.286 | 0.775 |  |
| April | -11.824 | 6.228 | -1.899 | 0.058 | . |
| May | -21.823 | 8.914 | -2.448 | 0.014 | * |
| June | -25.28 | 13.227 | -1.911 | 0.056 | . |
| July | -41.017 | 18.904 | -2.17 | 0.03 | * |
| August | -33.126 | 16.093 | -2.058 | 0.04 | * |
| September | -26.173 | 12.902 | -2.029 | 0.042 | * |
| October | -13.337 | 7.87 | -1.695 | 0.09 | . |
| November | -9.573 | 4.088 | -2.341 | 0.019 | * |
| December | 9.272 | 6.085 | 1.524 | 0.128 |  |
| Site 3 | 1.715 | 0.565 | 3.034 | 0.002 | ** |
| Site 4 | 1.001 | 0.717 | 1.396 | 0.163 |  |
| Site 7 | 2.402 | 0.85 | 2.827 | 0.005 | ** |
| Site 8 | 4.65 | 1.208 | 3.851 | 0 | *** |
| Temperature Maximum | -1.567 | 0.894 | -1.752 | 0.08 | . |
| Temperature Minimum | 4.131 | 1.282 | 3.222 | 0.001 | ** |
| Precipitation | 0.002 | 0.045 | 0.053 | 0.957 |  |
| Soil Moisture | -87.913 | 34.143 | -2.575 | 0.01 | * |
| NDVI | -37.371 | 24.598 | -1.519 | 0.129 |  |
| EVI | 71.024 | 39.265 | 1.809 | 0.07 | . |
| . = p < 0.1, * = p < 0.05, ** = p < 0.01, *** = p ≤ 0.001 | | | | |  |
