# Supplementary material for: Coccidioides undetected in soils from agricultural land and uncorrelated with time or the greater soil fungal community on undeveloped land
Source: PLoS Pathog. 2023 May 25;19(5):e1011391. doi: 10.1371/journal.ppat.1011391 (PMC10246812; doi:10.1371/journal.ppat.1011391)
Supplement: S4 Table — (DOCX) [file ppat.1011391.s010.docx]

**Table S4.** Pairwise differences between sites and, independently, between months by comparing estimated marginal means between logistic regression factor levels. Logistic regression calculated as positive *Coccidioides* detection using the CocciEnv qPCR assay, as a function of sampling site, sampling month and remotely sensed data. Only significant contrasts are shown. p-values are Tukey adjusted to total contrasts performed. Total month contrasts = 66. Total site contrasts = 10.

| Contrast | Estimate | Standard Error | z-value | p-value |  |
| --- | --- | --- | --- | --- | --- |
| Site 2 – Site 3 | -1.715 | 0.565 | -3.034 | 0.02 | * |
| Site 2 – Site 7 | -2.402 | 0.85 | -2.827 | 0.038 | * |
| Site 2 – Site 8 | -4.65 | 1.208 | -3.851 | 0.001 | ** |
| Site 4 – Site 7 | -3.649 | 1.086 | -3.359 | 0.007 | ** |
| Site 4 – Site 8 | -2.248 | 0.702 | -3.202 | 0.012 | * |
| Feb - Nov | 18.019 | 5.485 | 3.285 | 0.047 | * |
| May - Oct | -8.486 | 1.815 | -4.676 | 0.001 | *** |
| . = p < 0.1, * = p < 0.05, ** = p < 0.01, *** = p ≤ 0.001 | | | | |  |
