# Supplementary material for: Coccidioides undetected in soils from agricultural land and uncorrelated with time or the greater soil fungal community on undeveloped land
Source: PLoS Pathog. 2023 May 25;19(5):e1011391. doi: 10.1371/journal.ppat.1011391 (PMC10246812; doi:10.1371/journal.ppat.1011391)
Supplement: S5 Table — (DOCX) [file ppat.1011391.s011.docx]

**Table S5.** Coefficient tables (using the “glm” function) logistic regression models using a harmonic (A) or spline (B) term for month, showing *Coccidioides* detection, using the CocciEnv qPCR assay, as a function of sampling site and sampling month. n = 238. Comparison between models using harmonic, spline or categorical terms for sampling month.

| A. Harmonic Model | | | | |  |
| --- | --- | --- | --- | --- | --- |
|  | Estimate | Standard Error | z-value | p-value |  |
| Intercept | -1.045 | 0.313 | -3.334 | 0.001 | *** |
| sin(2·pi·month·12^-1^) | 0.303 | 0.213 | 1.426 | 0.154 |  |
| Site 3 | 2.091 | 0.451 | 4.638 | < 0.001 | *** |
| Site 4 | -0.185 | 0.479 | -0.386 | 0.699 |  |
| Site 7 | 0.688 | 0.441 | 1.561 | 0.119 |  |
| Site 8 | 1.097 | 0.426 | 2.576 | 0.010 | ** |
|  |  |  |  |  |  |
| B. Spline Model | | | | |  |
|  | Estimate | Standard Error | z-value | p-value |  |
| Intercept | -0.951 | 0.385 | -2.47 | 0.014 | * |
| Spline 1 | -0.732 | 0.692 | -1.058 | 0.290 |  |
| Spline 2 | 0.135 | 0.806 | 0.167 | 0.867 |  |
| Spline 3 | -0.39 | 0.466 | -0.838 | 0.402 |  |
| Site 3 | 2.081 | 0.451 | 4.614 | < 0.001 | *** |
| Site 4 | -0.189 | 0.479 | -0.395 | 0.693 |  |
| Site 7 | 0.683 | 0.441 | 1.548 | 0.122 |  |
| Site 8 | 1.086 | 0.425 | 2.552 | 0.011 | * |
|  |  |  |  |  |  |
| C. Model Comparison | | | | | |
|  | Parameters | AICc | Δ AICc | Model Likelihood | Log-Likelihood |
| Categorical | 16 | 290.72 | 0 | 1.0000 | -128.13 |
| Harmonic | 6 | 301.83 | 11.1 | 0.0039 | -144.73 |
| Spline | 8 | 306.39 | 15.66 | 0.0004 | -144.88 |
|  |  |  |  |  |  |
| * = p < 0.05, ** = p < 0.01, *** = p ≤ 0.001, df = degrees of freedom, AICc = AIC with small sample sizes (Burnham and Anderson 2002) | | | | | |
