# Supplementary material for: Coccidioides undetected in soils from agricultural land and uncorrelated with time or the greater soil fungal community on undeveloped land
Source: PLoS Pathog. 2023 May 25;19(5):e1011391. doi: 10.1371/journal.ppat.1011391 (PMC10246812; doi:10.1371/journal.ppat.1011391)
Supplement: S6 Table — (DOCX) [file ppat.1011391.s012.docx]

**Table S6.** PERMANOVA coefficient table (using the “adonis2” function) showing the ITS2 rDNA derived fungal community as a function of positive *Coccidioides* detection using the CocciEnv qPCR assay, sampling site, sampling month and remote sensing data. Permutations = 1000. n = 238.

|  | Degrees of Freedom | Sum of Squares | r^2^ | Pseudo-F | p-value |  |
| --- | --- | --- | --- | --- | --- | --- |
| *Coccidioides* Detection | 1 | 0.396 | 0.006 | 2.011 | 0.001 | *** |
| Site | 4 | 9.414 | 0.150 | 11.953 | 0.001 | *** |
| Month | 11 | 4.948 | 0.079 | 2.285 | 0.001 | *** |
| Temperature Maximum | 1 | 0.222 | 0.004 | 1.129 | 0.232 |  |
| Temperature Minimum | 1 | 0.194 | 0.003 | 0.986 | 0.466 |  |
| Precipitation | 1 | 0.224 | 0.004 | 1.135 | 0.221 |  |
| Soil Moisture | 1 | 0.21 | 0.003 | 1.066 | 0.324 |  |
| NDVI | 1 | 0.287 | 0.005 | 1.458 | 0.027 | * |
| EVI | 1 | 0.225 | 0.004 | 1.145 | 0.205 |  |
| *Coccidioides* Detection : Site | 4 | 1.06 | 0.017 | 1.346 | 0.004 | ** |
| *Coccidioides* Detection : Month | 11 | 2.423 | 0.039 | 1.118 | 0.039 | * |
| Site : Month | 44 | 11.228 | 0.179 | 1.296 | 0.001 | *** |
| *Coccidioides* Detection : Temperature Maximum | 1 | 0.146 | 0.002 | 0.740 | 0.946 |  |
| Site : Temperature Maximum | 4 | 0.802 | 0.013 | 1.018 | 0.422 |  |
| Month : Temperature Maximum | 11 | 2.199 | 0.035 | 1.015 | 0.386 |  |
| *Coccidioides* Detection : Temperature Minimum | 1 | 0.21 | 0.003 | 1.064 | 0.334 |  |
| Site : Temperature Minimum | 4 | 0.809 | 0.013 | 1.027 | 0.398 |  |
| Month : Temperature Minimum | 11 | 2.17 | 0.035 | 1.002 | 0.476 |  |
| Temperature Maximum : Temperature Minimum | 1 | 0.197 | 0.003 | 1.003 | 0.463 |  |
| *Coccidioides* Detection : Precipitation | 1 | 0.237 | 0.004 | 1.204 | 0.139 |  |
| Site : Precipitation | 4 | 0.718 | 0.011 | 0.911 | 0.812 |  |
| Month : Precipitation | 7 | 1.623 | 0.026 | 1.177 | 0.026 | * |
| Temperature Maximum : Precipitation | 1 | 0.293 | 0.005 | 1.486 | 0.027 | * |
| Temperature Minimum : Precipitation | 1 | 0.252 | 0.004 | 1.282 | 0.092 | . |
| *Coccidioides* Detection : Soil Moisture | 1 | 0.239 | 0.004 | 1.216 | 0.120 |  |
| Site : Soil Moisture | 4 | 0.791 | 0.013 | 1.005 | 0.441 |  |
| Month : Soil Moisture | 3 | 0.658 | 0.011 | 1.114 | 0.157 |  |
| Temperature Maximum : Soil Moisture | 1 | 0.241 | 0.004 | 1.222 | 0.119 |  |
| Temperature Minimum : Soil Moisture | 1 | 0.276 | 0.004 | 1.403 | 0.046 | . |
| Precipitation : Soil Moisture | 1 | 0.347 | 0.006 | 1.763 | 0.003 | ** |
| *Coccidioides* Detection : NDVI | 1 | 0.203 | 0.003 | 1.031 | 0.370 |  |
| Site : NDVI | 4 | 0.929 | 0.015 | 1.180 | 0.059 | . |
| Month : NDVI | 1 | 0.23 | 0.004 | 1.168 | 0.171 |  |
| Temperature Maximum : NDVI | 1 | 0.215 | 0.003 | 1.093 | 0.302 |  |
| Temperature Minimum : NDVI | 1 | 0.235 | 0.004 | 1.193 | 0.182 |  |
| Precipitation : NDVI | 1 | 0.234 | 0.004 | 1.189 | 0.149 |  |
| *Coccidioides* Detection : EVI | 1 | 0.212 | 0.003 | 1.078 | 0.289 |  |
| *Coccidioides* Detection : Site : Month | 11 | 2.086 | 0.033 | 0.963 | 0.693 |  |
| Site : Month : Soil Moisture : NDVI : EVI | 1 | 0.272 | 0.004 | 1.382 | 0.043 | * |
| Residual | 76 | 14.964 | 0.239 |  |  |  |
| **Total** | **237** | **62.62** | **1** |  |  |  |
| . = p < 0.1, * = p < 0.05, ** = p < 0.01, *** = p ≤ 0.001 | | | | | | |
