# Supplementary material for: Coccidioides undetected in soils from agricultural land and uncorrelated with time or the greater soil fungal community on undeveloped land
Source: PLoS Pathog. 2023 May 25;19(5):e1011391. doi: 10.1371/journal.ppat.1011391 (PMC10246812; doi:10.1371/journal.ppat.1011391)
Supplement: S7 Table — (DOCX) [file ppat.1011391.s013.docx]

**Table S7.** PERMANOVA coefficient table (using the “adonis2” function), with the 10 most abundant species removed, showing the ITS2 rDNA derived fungal community as a function of positive *Coccidioides* detection using the CocciEnv qPCR assay, sampling site, sampling month and remote sensing data. Permutations = 1000. n = 238.

|  | Degrees of Freedom | Sum of Squares | r^2^ | Pseudo-F | p-value |  |
| --- | --- | --- | --- | --- | --- | --- |
| *Coccidioides* Detection | 1 | 0.422 | 0.006 | 1.788 | 0.001 | *** |
| Site | 4 | 10.31 | 0.146 | 10.909 | 0.001 | *** |
| Month | 11 | 5.286 | 0.075 | 2.034 | 0.001 | *** |
| *Coccidioides* Detection : Site | 4 | 1.218 | 0.017 | 1.289 | 0.003 | ** |
| *Coccidioides* Detection : Month | 11 | 2.747 | 0.039 | 1.057 | 0.174 |  |
| Site : Month | 44 | 12.794 | 0.181 | 1.231 | 0.001 | *** |
| *Coccidioides* Detection : Site : Month | 25 | 5.465 | 0.077 | 0.925 | 0.957 |  |
| Residual | 137 | 32.368 | 0.458 |  |  |  |
| **Total** | **237** | **70.61** | **1** |  |  |  |
| . = p < 0.1, * = p < 0.05, ** = p < 0.01, *** = p ≤ 0.001 | | | | | | |
