# Supplementary material for: Coccidioides undetected in soils from agricultural land and uncorrelated with time or the greater soil fungal community on undeveloped land
Source: PLoS Pathog. 2023 May 25;19(5):e1011391. doi: 10.1371/journal.ppat.1011391 (PMC10246812; doi:10.1371/journal.ppat.1011391)
Supplement: S8 Table — (DOCX) [file ppat.1011391.s014.docx]

**Table S8.** PERMANOVA coefficient table (using the “adonis2” function), with the 50 most abundant species removed, showing the ITS2 rDNA derived fungal community as a function of positive *Coccidioides* detection using the CocciEnv qPCR assay, sampling site, sampling month and remote sensing data. Permutations = 1000. n = 238.

|  | Degrees of Freedom | Sum of Squares | r^2^ | Pseudo-F | p-value |  |
| --- | --- | --- | --- | --- | --- | --- |
| *Coccidioides* Detection | 1 | 0.451 | 0.005 | 1.483 | 0.019 | * |
| Site | 4 | 11.829 | 0.134 | 9.73 | 0.001 | *** |
| Month | 11 | 5.805 | 0.066 | 1.736 | 0.001 | *** |
| *Coccidioides* Detection : Site | 4 | 1.53 | 0.017 | 1.259 | 0.007 | ** |
| *Coccidioides* Detection : Month | 11 | 3.528 | 0.04 | 1.055 | 0.161 |  |
| Site : Month | 44 | 16.3 | 0.185 | 1.219 | 0.001 | *** |
| *Coccidioides* Detection : Site : Month | 25 | 7.173 | 0.081 | 0.944 | 0.948 |  |
| Residual | 137 | 41.642 | 0.472 |  |  |  |
| **Total** | **237** | **88.257** | **1** |  |  |  |
| . = p < 0.1, * = p < 0.05, ** = p < 0.01, *** = p ≤ 0.001 | | | | | | |
