# Supplementary material for: Coccidioides undetected in soils from agricultural land and uncorrelated with time or the greater soil fungal community on undeveloped land
Source: PLoS Pathog. 2023 May 25;19(5):e1011391. doi: 10.1371/journal.ppat.1011391 (PMC10246812; doi:10.1371/journal.ppat.1011391)
Supplement: S9 Table — (DOCX) [file ppat.1011391.s015.docx]

**Table S9.** Top 10 species removed. PERMANOVA coefficient table (using the “adonis2” function) showing the ITS2 rDNA derived fungal community as a function of *Coccidioides* detection using the CocciEnv qPCR assay, sampling site, sampling month and remote sensing data using the adonis2 function. Permutations = 1000. n = 238.

|  | Degrees of Freedom | Sum of Squares | r^2^ | Pseudo-F | p-value |  |
| --- | --- | --- | --- | --- | --- | --- |
| *Coccidioides* Detection | 1 | 0.422 | 0.006 | 1.855 | 0.003 | ** |
| Site | 4 | 10.31 | 0.146 | 11.32 | 0.001 | *** |
| Month | 11 | 5.286 | 0.075 | 2.11 | 0.001 | *** |
| Temperature Maximum | 1 | 0.263 | 0.004 | 1.154 | 0.203 |  |
| Temperature Minimum | 1 | 0.221 | 0.003 | 0.97 | 0.498 |  |
| Precipitation | 1 | 0.248 | 0.004 | 1.09 | 0.288 |  |
| Moisture | 1 | 0.246 | 0.003 | 1.082 | 0.307 |  |
| NDVI | 1 | 0.325 | 0.005 | 1.428 | 0.034 | * |
| EVI | 1 | 0.251 | 0.004 | 1.102 | 0.264 |  |
| *Coccidioides* Detection : Site | 4 | 1.194 | 0.017 | 1.311 | 0.003 | ** |
| *Coccidioides* Detection : Month | 11 | 2.727 | 0.039 | 1.089 | 0.089 | . |
| Site : Month | 44 | 12.658 | 0.179 | 1.263 | 0.001 | *** |
| *Coccidioides* Detection : Temperature Maximum | 1 | 0.163 | 0.002 | 0.718 | 0.955 |  |
| Site : Temperature Maximum | 4 | 0.939 | 0.013 | 1.031 | 0.351 |  |
| Month : Temperature Maximum | 11 | 2.512 | 0.036 | 1.003 | 0.466 |  |
| *Coccidioides* Detection : Temperature Minimum | 1 | 0.225 | 0.003 | 0.99 | 0.477 |  |
| Site : Temperature Minimum | 4 | 0.941 | 0.013 | 1.033 | 0.331 |  |
| Month : Temperature Minimum | 11 | 2.482 | 0.035 | 0.991 | 0.532 |  |
| Temperature Maximum : Temperature Minimum | 1 | 0.229 | 0.003 | 1.005 | 0.46 |  |
| *Coccidioides* Detection : Precipitation | 1 | 0.267 | 0.004 | 1.171 | 0.172 |  |
| Site : Precipitation | 4 | 0.827 | 0.012 | 0.908 | 0.828 |  |
| Month : Precipitation | 7 | 1.856 | 0.026 | 1.165 | 0.015 | * |
| Temperature Maximum : Precipitation | 1 | 0.323 | 0.005 | 1.418 | 0.027 | * |
| Temperature Minimum : Precipitation | 1 | 0.277 | 0.004 | 1.219 | 0.12 |  |
| *Coccidioides* Detection : Moisture | 1 | 0.262 | 0.004 | 1.152 | 0.174 |  |
| Site : Moisture | 4 | 0.884 | 0.013 | 0.971 | 0.592 |  |
| Month : Moisture | 3 | 0.743 | 0.011 | 1.087 | 0.209 |  |
| Temperature Maximum : Moisture | 1 | 0.273 | 0.004 | 1.2 | 0.125 |  |
| Temperature Minimum : Moisture | 1 | 0.307 | 0.004 | 1.35 | 0.041 | * |
| Precipitation : Moisture | 1 | 0.364 | 0.005 | 1.598 | 0.007 | ** |
| *Coccidioides* Detection : NDVI | 1 | 0.234 | 0.003 | 1.029 | 0.396 |  |
| Site : NDVI | 4 | 1.051 | 0.015 | 1.154 | 0.064 | . |
| Month : NDVI | 1 | 0.252 | 0.004 | 1.106 | 0.269 |  |
| Temperature Maximum : NDVI | 1 | 0.239 | 0.003 | 1.051 | 0.372 |  |
| Temperature Minimum : NDVI | 1 | 0.258 | 0.004 | 1.134 | 0.241 |  |
| Precipitation : NDVI | 1 | 0.252 | 0.004 | 1.107 | 0.264 |  |
| *Coccidioides* Detection : EVI | 1 | 0.253 | 0.004 | 1.11 | 0.268 |  |
| *Coccidioides* Detection : Site : Month | 11 | 2.438 | 0.035 | 0.973 | 0.651 |  |
| Site : Month : Moisture : NDVI : EVI | 1 | 0.302 | 0.004 | 1.327 | 0.041 | * |
| Residual | 76 | 17.304 | 0.245 |  |  |  |
| **Total** | **237** | **70.61** | **1** |  |  |  |
| . = p < 0.1, * = p < 0.05, ** = p < 0.01, *** = p ≤ 0.001 | | | | | | |
