# Supplementary material for: Coccidioides undetected in soils from agricultural land and uncorrelated with time or the greater soil fungal community on undeveloped land
Source: PLoS Pathog. 2023 May 25;19(5):e1011391. doi: 10.1371/journal.ppat.1011391 (PMC10246812; doi:10.1371/journal.ppat.1011391)
Supplement: S10 Table — (DOCX) [file ppat.1011391.s016.docx]

**Table S10.** Top 50 species removed. PERMANOVA coefficient table (using the “adonis2” function) showing the ITS2 rDNA derived fungal community as a function of *Coccidioides* detection using the CocciEnv qPCR assay, sampling site, sampling month and remote sensing data using the adonis2 function. Permutations = 1000. n = 238.

|  | Degrees of Freedom | Sum of Squares | r^2^ | Pseudo-F | p-value |  |
| --- | --- | --- | --- | --- | --- | --- |
| *Coccidioides* Detection | 1 | 0.451 | 0.005 | 1.515 | 0.009 | ** |
| Site | 4 | 11.829 | 0.134 | 9.939 | 0.001 | *** |
| Month | 11 | 5.805 | 0.066 | 1.773 | 0.001 | *** |
| Temperature Maximum | 1 | 0.295 | 0.003 | 0.991 | 0.451 |  |
| Temperature Minimum | 1 | 0.286 | 0.003 | 0.961 | 0.527 |  |
| Precipitation | 1 | 0.335 | 0.004 | 1.127 | 0.208 |  |
| Moisture | 1 | 0.321 | 0.004 | 1.078 | 0.286 |  |
| NDVI | 1 | 0.462 | 0.005 | 1.554 | 0.007 | ** |
| EVI | 1 | 0.356 | 0.004 | 1.197 | 0.127 |  |
| *Coccidioides* Detection : Site | 4 | 1.498 | 0.017 | 1.259 | 0.008 | ** |
| *Coccidioides* Detection : Month | 11 | 3.507 | 0.04 | 1.071 | 0.111 |  |
| Site : Month | 44 | 16.056 | 0.182 | 1.226 | 0.001 | *** |
| *Coccidioides* Detection : Temperature Maximum | 1 | 0.212 | 0.002 | 0.712 | 0.964 |  |
| Site : Temperature Maximum | 4 | 1.234 | 0.014 | 1.037 | 0.341 |  |
| Month : Temperature Maximum | 11 | 3.294 | 0.037 | 1.006 | 0.442 |  |
| *Coccidioides* Detection : Temperature Minimum | 1 | 0.334 | 0.004 | 1.122 | 0.239 |  |
| Site : Temperature Minimum | 4 | 1.188 | 0.013 | 0.998 | 0.474 |  |
| Month : Temperature Minimum | 11 | 3.194 | 0.036 | 0.976 | 0.662 |  |
| Temperature Maximum : Temperature Minimum | 1 | 0.308 | 0.003 | 1.034 | 0.379 |  |
| *Coccidioides* Detection : Precipitation | 1 | 0.324 | 0.004 | 1.09 | 0.292 |  |
| Site : Precipitation | 4 | 1.06 | 0.012 | 0.89 | 0.883 |  |
| Month : Precipitation | 7 | 2.341 | 0.027 | 1.124 | 0.052 | . |
| Temperature Maximum : Precipitation | 1 | 0.403 | 0.005 | 1.353 | 0.047 | * |
| Temperature Minimum : Precipitation | 1 | 0.352 | 0.004 | 1.183 | 0.15 |  |
| *Coccidioides* Detection : Moisture | 1 | 0.331 | 0.004 | 1.113 | 0.253 |  |
| Site : Moisture | 4 | 1.143 | 0.013 | 0.96 | 0.68 |  |
| Month : Moisture | 3 | 0.952 | 0.011 | 1.066 | 0.233 |  |
| Temperature Maximum : Moisture | 1 | 0.372 | 0.004 | 1.25 | 0.098 | . |
| Temperature Minimum : Moisture | 1 | 0.339 | 0.004 | 1.141 | 0.224 |  |
| Precipitation : Moisture | 1 | 0.404 | 0.005 | 1.358 | 0.03 | * |
| *Coccidioides* Detection : NDVI | 1 | 0.325 | 0.004 | 1.092 | 0.255 |  |
| Site : NDVI | 4 | 1.307 | 0.015 | 1.098 | 0.131 |  |
| Month : NDVI | 1 | 0.315 | 0.004 | 1.058 | 0.358 |  |
| Temperature Maximum : NDVI | 1 | 0.274 | 0.003 | 0.919 | 0.657 |  |
| Temperature Minimum : NDVI | 1 | 0.297 | 0.003 | 0.999 | 0.495 |  |
| Precipitation : NDVI | 1 | 0.301 | 0.003 | 1.013 | 0.44 |  |
| *Coccidioides* Detection : EVI | 1 | 0.351 | 0.004 | 1.179 | 0.145 |  |
| *Coccidioides* Detection : Site : Month | 11 | 3.099 | 0.035 | 0.947 | 0.818 |  |
| Site : Month : Moisture : NDVI : EVI | 1 | 0.389 | 0.004 | 1.307 | 0.017 | * |
| Residual | 76 | 22.614 | 0.256 |  |  |  |
| **Total** | **237** | **88.257** | **1** |  |  |  |
| . = p < 0.1, * = p < 0.05, ** = p < 0.01, *** = p ≤ 0.001 | | | | | | |
