# Supplementary material for: Coccidioides undetected in soils from agricultural land and uncorrelated with time or the greater soil fungal community on undeveloped land
Source: PLoS Pathog. 2023 May 25;19(5):e1011391. doi: 10.1371/journal.ppat.1011391 (PMC10246812; doi:10.1371/journal.ppat.1011391)
Supplement: S11 Table — (DOCX) [file ppat.1011391.s017.docx]

**Table S11:** Consolidated indicator species for *Coccidioides* positive and *Coccidioides* negative rodent burrow soil samples. Significance was calculated with 1000 permutations using the “multipatt” command with indicspecies version 1.7.12. All likely alternative species candidates were identified and included (highest % match) via NCBI Nucleotide Blast (https://blast.ncbi.nlm.nih.gov/) for each prospective indicator species. IndVal and p-value are applicable to all alternative species candidates within each subsection. n = 238.

|  | *Coccidioides* positive samples (n = 89) | | | | | |  |
| --- | --- | --- | --- | --- | --- | --- | --- |
|  | | BLASTN % | Reads | IndVal | p-value | |  |
| *Aspergillus penicillioides* | | 98.9 | 20045 | 0.610 | 0.001 | | *** |
| *Aspergillus hordei* | | 98.5 |  |  |  | |  |
| *Acremonium charticola* | | 100.0 | 5993 | 0.478 | 0.002 | | ** |
| *Acremonium tubakii* | | 100.0 |  |  |  | |  |
| *Acremonium acutatum* | | 99.0 |  |  |  | |  |
| *Fusarium sp.* | | 99.7 | 1130 | 0.359 | 0.002 | | ** |
| *Bifusarium domesticum* | | 99.7 |  |  |  | |  |
| *Bifusarium delphinoides* | | 99.7 |  |  |  | |  |
| *Bifusarium lunatum* | | 98.5 |  |  |  | |  |
| *Tricellula inaequalis* | | 96.3 | 1213 | 0.296 | 0.003 | | ** |
| *Tulostoma pseudopulchellum* | | 95.9 | 1753 | 0.279 | 0.005 | | ** |
| *Simplicillium album* | | 99.7 | 317 | 0.257 | 0.009 | | ** |
| *Neostagonosporea sp.* | | 100.0 | 316 | 0.249 | 0.024 | | * |
| *Septoria arundinacea* | | 99.7 |  |  |  | |  |
| *Neostagonosporea elegiae* | | 98.8 |  |  |  | |  |
| *Coccidioides immitis* | | 99.7 | 285 | 0.212 | 0.022 | | * |
|  | |  |  |  |  | |  |
|  | *Coccidioides* negative samples (n = 149) | | | | | |  |
|  | | BLASTN % | Reads | IndVal | p-value | |  |
| *Gaertneriomyces semiglobifer* | | 96.4 | 812 | 0.393 | 0.006 | | ** |
| *Gaertneriomyces californicus* | | 96.1 |  |  |  | |  |
| *Spizellomyces acuminatus* | | 99.5 | 1037 | 0.374 | 0.011 | | * |
|  | |  |  |  |  | |  |
| * = p < 0.05, ** = p < 0.01, *** = p ≤ 0.001, IndVal = indicator value | | | | | |  | |
